# Aptamer‐Conjugated Exosomes Ameliorate Diabetes‐Induced Muscle Atrophy by Enhancing SIRT1/FoxO1/3a‐Mediated Mitochondrial Function

**DOI:** 10.1002/jcsm.13717

**Published:** 2025-01-28

**Authors:** Jia Song, Mengmeng Yang, Longqing Xia, Liming Wang, Kewei Wang, Yingyue Xiang, Jun Cheng, Jun Chen, Jidong Liu, Ruxing Zhao, Fuqiang Liu, Zheng Sun, Xinguo Hou, Nan Zang, Li Chen

**Affiliations:** ^1^ Department of Endocrinology and Metabolism Qilu Hospital of Shandong University Jinan Shandong China; ^2^ Shandong Provincial Key Laboratory of Spatiotemporal Regulation and Precision Intervention in Endocrine and Metabolic Diseases, Shandong Provincial Engineering Research Center for Advanced Technologies in Prevention and Treatment of Chromic Metabolic Diseases, Institute of Endocrine and Metabolic Diseases of Shandong University Jinan Shandong China; ^3^ Department of Clinical Laboratory, Shandong Engineering & Technology Research Center for Tumor Marker Detection The Second Hospital of Shandong University Jinan Shandong China

**Keywords:** Apt, mitochondrial function, MSC‐EXOs, muscle atrophy, SIRT1/FoxO1/3a

## Abstract

**Background:**

Muscle atrophy is associated with Type 2 diabetes mellitus, which reduces the quality of life and lacks effective treatment strategies. Previously, it was determined that human umbilical cord mesenchymal stromal cell (hucMSC)–derived exosomes (EXOs) ameliorate diabetes‐induced muscle atrophy. However, the systemic application of EXOs is less selective for diseased tissues, which reduces their efficacy and safety associated with their nonspecific biological distribution in vivo. Therefore, improving exosomal targeting is imperative. In this study, a skeletal muscle–specific aptamer (Apt) was used to explore the effects of Apt‐functionalized EXOs derived from hucMSCs in diabetes‐associated muscle atrophy and its specific mechanisms.

**Methods:**

Diabetic db/db mice and C2C12 myotubes were used to explore the effects of MSC‐EXOs or Apt‐EXOs in alleviating muscle atrophy. Grip strength, muscle weight and muscle fibre cross‐sectional area (CSA) were used to evaluate skeletal muscle strength and muscle mass. Western blot analysis of muscle atrophy signalling, including MuRF1 and Atrogin 1 and the mitochondrial complex and Seahorse analysis were performed to investigate the underlying mechanisms of MSC‐EXOs or Apt‐EXOs on muscle atrophy.

**Results:**

MSC‐EXOs increased grip strength (*p* = 0.0002) and muscle mass (*p* = 0.0044 for tibialis anterior (TA) muscle, *p* = 0.002 for soleus (SO) muscle) in db/db mice. It also increased the CSA of muscle fibres (*p* = 0.0011 for all fibres, *p* = 0.0036 for slow muscle fibres and *p* = 0.0089 for fast muscle fibres) and the percentage of slow‐to‐fast muscle fibres (*p* = 0.0109). However, Atrogin 1 (*p* = 0.0455) and MuRF1 expression (*p* = 0.0168) was reduced. MSC‐EXOs activated SIRT1/FoxO1/3a signalling and enhanced mitochondrial function in db/db mice and C2C12 myotubes. SIRT1 knockdown decreased the beneficial antiatrophic effects of MSC‐EXOs. Additionally, Apt conjugation increased the effect of MSC‐EXOs on muscle atrophy and myofiber‐type transition (*p* = 0.0133 for grip strength, *p* = 0.0124 for TA muscle weight, *p* = 0.0008 for SO muscle weight, *p* < 0.0001 for CSA of all muscle fibres, *p* = 0.0198 for CSA of slow muscle fibres, *p* = 0.0213 for CSA of fast muscle fibres, *p* = 0.011 for percentage of slow–fast muscle fibres, *p* = 0.0141 for Atrogin 1 expression and *p* = 0.005 for MuRF1 expression).

**Conclusions:**

The results suggest that hucMSC‐derived exosomes ameliorate diabetes‐associated muscle atrophy by enhancing SIRT1/FoxO1/3a‐mediated mitochondrial function and that Apt conjugation strengthens the effects of MSC‐EXOs on muscle atrophy. These findings demonstrate the therapeutic potential of muscle‐targeted MSC‐EXOs for the treatment of muscle atrophy.

## 1 Introduction

Muscle atrophy is characterized by the progressive loss of muscle mass and leads to decreased muscle function. It is closely associated with aging (also known as sarcopenia), immobility and chronic diseases such as obesity and Type 2 diabetes mellitus (T2DM) [1]. The loss of muscle function results in limited activity, reduced quality of life and increased mortality in patients with T2DM muscular disorders, while there is no specific treatment [2, 3]. Therefore, it is of vital importance to explore the mechanisms and effective strategies of muscle atrophy.

In skeletal muscle, there exists two major types of muscle fibres called slow‐twitch oxidative (Type I) fibres and fast‐twitch glycolytic (Type II) fibres. Compared with fast‐glycolytic fibres, slow‐oxidative fibres exhibit more mitochondrial density and produce abundant ATP through oxidative phosphorylation (OXPHOS), which can improve exercise endurance [4, 5]. The conversion of muscle fibre type from slow oxidative to fast glycolytic leads to the decrease in oxidative metabolism, which is accompanied by the reduction in mitochondrial contents and the endurance muscle function [3]. Mitochondrial dysfunction plays an important role in muscle atrophy [6]. Impaired mitochondrial function and abnormal mitochondrial accumulation in aged mice can lead to muscle atrophy [7, 8]. Disorders of glucose and lipid metabolism, insulin resistance and inflammatory responses in diabetes lead to mitochondrial dysfunction in skeletal muscles, which is accompanied by abnormal myofiber‐type transition and muscle atrophy [9, 10] (S1).

Forkhead box O (FoxO) family members are key transcription factors that increase muscle atrophy, which translocate into the nucleus and promote the expression of the E3 ubiquitin ligases MuRF1 and Atrogin 1 after dephosphorylation and/or acetylation [11] (S2). FoxO1/3/4 deletion enhances the function of muscle mitochondria and increases the lean mass [12], whereas FoxO3 activation causes expression of the MuRF1 and Atrogin 1 and profound loss of muscle mass [13]. Sirtuins, particularly sirtuin 1 (SIRT1), are associated with metabolic diseases such as obesity and diabetes. SIRT1, a nicotinamide adenine dinucleotide (NAD^+^)–dependent histone deacetylase, is crucial in skeletal muscle remodelling by inhibiting FoxO transcriptional activity [14]. SIRT1 activation in skeletal muscles of diabetic mice promotes mitochondrial biosynthesis [15].

Mesenchymal stromal cell (MSC)–based therapies have broad prospects for alleviating diabetes and its complications through paracrine actions [16] (S3). Mesenchymal stromal cell–derived exosomes (MSC‐EXOs) contain various bioactive molecules that mediate the therapeutic effects of MSCs [17]. Our previous study reported that human umbilical cord mesenchymal stromal cells (hucMSCs) ameliorate diabetes‐induced muscle atrophy via exosomes (EXOs) [18]. Compared with MSCs, EXOs are easier to obtain, have lower immunogenicity and tumorigenicity and exhibit innate stability and biocompatibility in vivo, making them an ideal choice for regenerative therapy [19] (S4, S5). However, the systemic application of EXOs is less selective for diseased tissues, which reduces their efficacy and safety associated with nonspecific biological distribution in vivo [20]. Therefore, improving exosomal targeting is a promising strategy. Aptamers (Apts) screened by systematic evolution of ligands by exponential enrichment (SELEX) are single‐stranded DNA/RNA oligonucleotides capable of forming three‐dimensional structures and binding target molecules with high affinity and specificity, which have been used to identify diseased tissues in a covalent or physical coupling manner [21] (S6). Apt‐functionalized exosomes (Apt‐EXOs) have been reported in cancer and osteoporosis treatment [22, 23]. Their ability to recognise and effectively bind to target molecules has prompted this investigation to determine if Apts could promote the preferential recruitment of EXOs to skeletal muscles and enhance their efficacy in muscle atrophy.

Therefore, the aim of this study is to investigate the selectivity of a previously selected skeletal muscle–specific Apt [24] combined with EXOs derived from hucMSCs on diabetes‐associated muscle atrophy and determine its specific underlying mechanisms.

## 2 Materials and Methods

### 2.1 Human MSC Isolation and Characterisation

With informed parental consent, hucMSCs were obtained from the fresh umbilical cords of healthy newborns, as reported previously [18]. The study was approved by the Ethics Committee at Qilu Hospital of Shandong University. hucMSCs were cultured in α‐MEM (Gibco, New York, United States) containing 10% foetal bovine serum (FBS; Gibco), 100 U/mL penicillin and 100 μg/mL streptomycin (Gibco). Flow cytometry analysis was used to identify hucMSCs that positively expressed CD105 (Cat. No. 17111‐80; BioGems, California, United States) and CD73 (Cat. No. 05811‐60; BioGems) and did not express CD34 (Cat. No. 343505; BioLegend, California, United States) and HLA‐DR (Cat. No. 327021; BioLegend). To detect the differentiation potential of hucMSCs, the cells were cultured in adipogenic (Cat. No. HUXUC‐90031; OriCell, China), osteogenic (Cat. No. HUXUC‐90021; OriCell) and chondrogenic (Cat. No. HUXUC‐90041; OriCell) differentiation medium. Oil Red O (Cat. No. G1262; Solarbio, China), Alizarin Red S (Cat. No. G1450; Solarbio) and Alcian Blue (Cat. No. ALCB‐10001; OriCell) were used for staining lipid droplets, calcium nodes and the chondrosphere.

### 2.2 EXO Isolation and Characterisation

hucMSCs or human embryo lung fibroblasts (HELFs) were seeded in ordinary culture flasks. Following 48 h of culture in the EXO‐free medium when they reached 90% confluency, the hucMSC‐ and HELF‐conditioned mediums were pooled. The pooled mediums were centrifuged at 10000 × *g* for 1 h and filtered through a 0.22‐μm filter to remove cellular debris and large vesicles. Subsequently, the medium was ultracentrifuged at 120 000 × *g* for 70 min at 4°C to obtain EXO pellets that were resuspended in PBS. The protein contents of the isolated EXOs were determined using a BCA protein assay kit (Beyotime, China). The morphology of the EXOs was observed using a transmission electron microscope (TEM; FEI Tecnai G2 Spirit; FEI company, Oregon, United States). The size of the EXOs were quantified using NanoSight tracking analysis (NTA; Particle Metrix, Germany). Western blot was used to detect the expression of exosomal markers CD9, TSG101, HSP70 and calnexin.

### 2.3 Cell Culture and RNAi

HELFs were obtained from the China Cell Culture Center (Shanghai, China). The mouse podocyte cell line MPC5 and monocyte/macrophage cell line RAW264.7 were purchased from Mingjing Biology (Shanghai, China) and the agent of American Type Culture Collection (ATCC, Virginia, United States), respectively. HELFs, MPC5 and RAW264.7 were all cultured in high‐glucose Dulbecco's modified Eagle's medium (DMEM, Gibco) supplemented with 10% FBS, 100 U/mL penicillin and 100 μg/mL streptomycin at 37°C in a 5% CO_2_ incubator. The alpha mouse liver 12 (AML12) cell line was obtained from Shanghai Cell Bank (China), cultured in DMEM/F12 medium (Gibco) containing 10% FBS, 100 U/mL penicillin, 100 μg/mL streptomycin, 1% insulin–transferrin–selenium and 0.1 μmol/L dexamethasone.

Mouse C2C12 myoblasts were purchased from China Infrastructure of Cell Line Resource (Beijing, China) and cultured in high‐glucose DMEM containing 10% FBS and antibiotics. A differentiation medium consisting of DMEM plus 2% heat‐inactivated horse serum and antibiotics was introduced for 4 days after reaching 80%–90% confluency. The medium was refreshed every 2 days. The fully differentiated myotubes were stimulated with palmitate (PA, 0.6 mM) and MSC‐EXOs (25 μg/mL) or HELF‐EXOs (25 μg/mL) for 24 h. After intervention, myotube images were captured with a microscope (BX53; Olympus, Japan) and the camera software cellSens Standard was used to measure the transverse diameter at the widest point of each myotube. To further investigate the mechanism, myotubes were transfected with small interfering RNA (siRNA) before MSC‐EXO treatment using Lipofectamine 2000 transfection reagent (Invitrogen, California, United States) following the manufacturer's instructions. Briefly, C2C12 myoblasts were plated in six‐well plates and fully differentiated before being switched to Opti‐MEM I‐reduced serum medium (Gibco) containing SIRT1 siRNA (125 nM) for 6 h. Subsequently, the culture medium was replaced with the differentiation medium, and myotubes were treated with MSC‐EXOs for 24 h. The siRNA oligonucleotides were synthesised by GenePharma Co. Ltd. (Shanghai, China). The sequences of siRNA for the negative control (NC) were as follows: sense 5′‐UUCUCCGAACGUGUCACGUTT‐3′ and antisense 5′‐ACGUGACACGUUCGGAGAATT‐3′. The sequences for SIRT1 siRNA were as follows: sense 5′‐GGGAUCAAGAGGUUGUUAATT‐3′ and antisense 5′‐UUAACAACCUCUUGAUCCCTT‐3′.

Additionally, to further confirm the role of SIRT1 in MSC‐EXOs, C2C12 myoblasts were also transfected with short hairpin RNA (shRNA, GenePharma Co. Ltd.) using lentivirus following the manufacturer's instructions. Next, they were fully differentiated for 4 days and treated with MSC‐EXOs for 24 h. The shRNA oligonucleotides were synthesised by GenePharma Co. Ltd. The sequences of shRNA for NC were as follows: 5′‐TTCTCCGAACGTGTCACGT‐3′. The sequences for SIRT1 shRNA were as follows: 5′‐TTAACAACCTCTTGATCCC‐3′.

### 2.4 Apt Formation

Apt oligonucleotides were synthesised by Sangon Biotech Co. Ltd. (Shanghai, China). The sequences were as follows: 5′‐CAGGAGCCGAGAACCGGTTGGTGGGTAATCCTGTTAGCGC‐3′, which was identified by a cell‐internalisation SELEX approach using C2C12 myoblasts as it preferentially internalises in skeletal muscle cells and exhibits decreased affinity for off‐target cells [24]. A random sequence with the same number of bases served as a control. Oligonucleotides were resuspended in Tris‐EDTA (TE) buffer (pH 8.0; Sangon Biotech, Shanghai, China) at a final concentration of 100 μM. Subsequently, a folding buffer (DPBS [pH 7.5] containing 1 mM MgCl_2_) was used to form secondary structures. Folding conditions were as follows: 10 min at 70°C, snap‐cooling on ice for 5 min and slow cooling at 37°C for 30 min. The RNAstructure website was used to predict the secondary structure of the Apts (https://rna.urmc.rochester.edu/RNAstructureWeb/index.html).

### 2.5 Confirmation of Apt Affinity

To confirm the affinity of the Apt for C2C12, the suspended C2C12, AML12 cell line, mouse podocyte line MPC5 and mouse monocyte/macrophage RAW264.7 were reacted with 200 nM Cy3‐labelled Apts or random sequence. After removing the excess Apts by centrifugation, flow cytometry was used to determine the percentage of Cy3‐positive cells. To observe the conjugation conditions of Apts and the above‐mentioned cells, 200 nM of Cy3‐labelled Apts or random were added to cultured cells at 37°C for 3 h. After washing three times with PBS and staining with DAPI, the signals of the conjugated Apts were captured using a fluorescence microscope (BX53; Olympus).

To confirm the specificity of the Apt for skeletal muscles, 2.5 μM Cy3‐labelled Apts or random was dissolved in 300 μL folding buffer and was injected into mice via tail vein. Thirty minutes after administration, the mice were sacrificed, and their heart, liver, spleen, lungs, kidneys and skeletal muscles were dissected and scanned using an in vivo imaging system (IVIS; Tanon ABL X5, China).

### 2.6 Conjugation of Apts and EXOs

The 5′‐end of the Apt was modified with an aldehyde group, which could react with an amino group on the membrane of MSC‐EXOs via the Schiff base reaction. Specifically, 200 nM aldehyde‐modified Apts and 1.0 mg/mL MSC‐EXOs in PBS were incubated in a rotating mixer overnight at 4°C. The unconjugated Apts were removed by centrifugation in 100‐kDa ultrafiltration tubes. Apt‐EXOs were prepared for the animal experiments.

### 2.7 Animal Experimentation

All animal experiments were approved by the Animal Ethics Committee of Qilu Hospital of Shandong University. Four‐week‐old male db/db (a widely used spontaneous T2DM model) and db/m (control) mice were purchased from Jiangsu Huachuang Sino PharmaTech Co. Ltd. (Suzhou, China) and fed a normal chow diet. The mice were housed in a 12‐h light/dark cycle at 22°C –25°C with 55% ± 5% humidity. After 1 week of adaptive feeding, T2DM was defined as fasting glucose ≥ 16.7 mmol/L twice in succession. Then, 200 μg MSC‐ or Apt‐EXOs were suspended in PBS and injected into db/db mice (5‐week‐old) via the tail vein every 3 days for 6 weeks.

### 2.8 Exosomal Tracing

For in vivo tracing, MSC‐ or Apt‐EXOs were labelled with DIR (Invitrogen) and injected into mice via the tail vein. The mice and their harvested organs were scanned using an IVIS, 6 h after administration. For in vitro tracing, MSC‐ or Apt‐EXOs were labelled with the PKH67 Green Fluorescent Cell Linker Kit (PKH67, Sigma‐Aldrich, Missouri, United States) and incubated with fully differentiated myotubes for 3 h. Fluorescence signals were captured using a fluorescence microscope (BX53; Olympus).

### 2.9 Metabolic Testing and In Vivo Muscle Performance Analysis

Intraperitoneal glucose tolerance test (IPGTT) and intraperitoneal insulin tolerance test (IPITT) were performed as previously described [18]. Forelimb grip strength was measured using an electronic dynamometer (Handpi HP‐5N, Beijing, China). The mice were trained to grasp the horizontal bar connected to the dynamometer with their forelimbs and gently pull backward in the horizontal direction. The force applied to the bar each time before the mouse lost its grip was recorded. Each mouse was assessed three times, and the measured values were averaged.

### 2.10 Histology and Immunohistochemical Staining

One week after metabolic testing and in vivo muscle performance analysis, the mice were anaesthetised with CO_2_ and decapitated. Bilateral tibialis anterior (TA), soleus (SO) and gastrocnemius (GAS) muscles were dissected. TA and SO muscle weights were normalized to body weight. One muscle was frozen at −80°C after liquid nitrogen treatment, and the other was fixed in 4% paraformaldehyde for haematoxylin–eosin (H&E) and immunohistochemical staining. The fixed TA and GAS muscles were embedded in paraffin and were sliced to a 5‐μm thickness at the maximum cross‐section. After deparaffinisation, the slides were stained with H&E following standard procedures. Immunohistochemical staining was performed as described previously [18]. Briefly, antigen retrieval was performed using an antigen unmasking buffer after the slides were deparaffinized. Hydrogen peroxide (3%) was used to inactivate the endogenous peroxidases for 15 min, followed by 30 min of blocking in a protein‐blocking solution (10% normal goat serum) at room temperature. The slides were then incubated with anti–Fast Myosin Skeletal Heavy Chain (1:1000; Cat. No. ab91506; Abcam, Massachusetts, United States) and anti–Slow Myosin Skeletal Heavy Chain antibodies (1:1000; Cat. No. ab234431; Abcam) at 4°C overnight. After incubation with the secondary antibody at room temperature for 60 min, the slides were stained with 3,3′‐diaminobenzidine (DAB) solution. Images were acquired using a microscope (BX53; Olympus). Image‐Pro Plus software was used to calculate the cross‐sectional areas (CSAs) of the muscle fibres.

### 2.11 Western Blot

TA muscle, C2C12 myotubes and EXOs were lysed in radioimmunoprecipitation assay (RIPA) lysis buffer (P0013B; Beyotime, Shanghai, China), and the proteins were separated and transferred onto polyvinylidene difluoride (PVDF) membranes (IPVH00010 0.45 μm; Millipore, Massachusetts, United States). After blocking with 5% skim milk for 1 h at room temperature, the membranes were incubated with specific primary antibodies at 4°C overnight. After incubation with horseradish peroxidase–conjugated secondary antibodies at room temperature for 1 h, the proteins were detected using enhanced chemiluminescence. Quantification of bands was performed using ImageJ software and was normalized by using GAPDH as a control.

The following primary antibodies were used: Atrogin 1 (1:5000; Cat. No. 67172‐1‐Ig; Proteintech, China); MuRF1 (1:1000; Cat. No. 55456‐1‐AP; Proteintech); GAPDH (1:5000; Cat. No. AB0037; Abways, China); SIRT1 (1:1000; Cat. No. 13161‐1‐AP; Proteintech); forkhead box O1 (FoxO1; 1:1000; Cat. No. 2880S; CST); phosphorylated‐(p‐)FoxO1(Ser319) (1:500; Cat. No. AP1090; ABclonal, China); forkhead box O3a (FoxO3a; 1:1000; Cat. No. 2497S; CST); p‐FoxO3a(Ser253) (1:500; Cat. No. bs‐3140R; Bioss); NADH dehydrogenase (ubiquinone) 1 beta subcomplex 8 (NDUFB8; 1:1000; Cat. No. 14794‐1‐AP; Proteintech); succinate dehydrogenase complex subunit B (SDHB; 1:5000; Cat. No. 10620‐1‐AP; Proteintech); ubiquinol‐cytochrome c reductase core protein II (UQCRC2; 1:1000; Cat. No. 14742‐1‐AP; Proteintech); cytochrome c oxidase II (MTCO2; 1:1000; Cat. No. 55070‐1‐AP; Proteintech); ATP synthase, H+ transport, mitochondrial F1 complex, alpha subunit 1 (ATP5A1; 1:2000; Cat. No. 14676‐1‐AP; Proteintech); heat shock protein 90 (HSP90; 1:1000; Cat. No. A5027; ABclonal, China); myogenic differentiation 1 (MyoD1; 1:1000; Cat. No. 18943‐1‐AP; Proteintech); CD9 (1:1000; Cat. No. ab263019; Abcam); TSG101 (1:1000; Cat. No. ab125011; Abcam); HSP70 (1:1000; Cat. No. ab181606; Abcam); and calnexin (1:1000; Cat. No. CY5839; Abways).

### 2.12 Real‐Time Quantitative PCR Analysis

Total RNA from TA muscles was extracted using Trizol reagent (Invitrogen) following the manufacturer's procedure. Then, 1 μg RNA was reverse‐transcribed into cDNA using the Prime Script RT Reagent Kit (Cat. No. RR047A; Takara, Japan). Primers were chemically synthesised by GenePharma Co. Ltd. The primer sequences were as follows: *Gapdh*, sense 5′‐AAGGGCTCATGACCACAGTC‐3′ and antisense 5′‐CAGGGATGATGTTCTGGGCA‐3′; *SIRT1*, sense 5′‐GCTGACGACTTCGACGACG‐3′ and antisense 5′‐TCGGTCAACAGGAGGTTGTCT‐ 3′; *FoxO1*, sense 5′‐CCCAGGCCGGAGTTTAACC‐3′ and antisense 5′‐GTTGCTCATAAAGTCGGTGCT‐3′; *FoxO3*, sense 5′‐CTGGGGGAACCTGTCCTATG‐3′ and antisense 5′‐TCATTCTGAACGCGCATGAAG‐3′; *MyHC I*, sense 5′‐CTTCTACAGGCCTGGGCTTAC‐3′ and antisense 5′‐CTCCTTCTCAGACTTCCGCAG‐3′; *MyHC IIa*, sense 5′‐TTCCAGAAGCCTAAGGTGGTC‐3′ and antisense 5′‐GCCAGCCAGTGATGTTGTAAT‐3′; *MyHC IIb*, sense 5′‐CTTGTCTGACTCAAGCCTGCC‐3′ and antisense 5′‐TCGCTCCTTTTCAGACTTCCG‐3′; *Myoglobin*, sense 5′‐GAAGAAGCATGGTTGCACCG‐3′ and antisense 5′‐TCCAGGTACTTGACCGGGAT‐3′; *Tnni1*, sense 5′‐ATGCCGGAAGTTGAGAGGAAA‐3′ and antisense 5′‐TCCGAGAGGTAACGCACCTT‐3′; and *Tnnt1*, sense 5′‐CCTGTGGTGCCTCCTTTGATT‐3′ and antisense 5′‐TGCGGTCTTTTAGGCAATGAG‐3′. Real‐time PCR was conducted with the SYBR Green PCR Kit (Cat. No. RR420A; Takara); gene expression changes were determined with the comparative CT (2^−ΔΔCt^) method, and quantification was achieved by normalization using *Gapdh* as the control.

### 2.13 TEM

TA muscles were dissected and rapidly fixed in 2.5% glutaraldehyde and 1% phosphate‐buffered osmium tetroxide. After embedding, sectioning and double staining with uranyl acetate and lead citrate, electron photomicrographs of the TA muscle ultrastructure were captured using TEM (JEM‐1200EX II, JEOL, Tokyo, Japan). The cellSens Standard software was used to measure the size of intermyofibrillar (IMF) mitochondria. And the quantity of IMF mitochondria was presented as the total number of mitochondria divided by the number of sarcomeric Z‐lines.

### 2.14 Succinate Dehydrogenase (SDH) and Lactate Dehydrogenase (LDH) Activity Assay

The GAS muscle and C2C12 myotubes were used to detect SDH and LDH activities using the SDH (Cat. No. BC0955; Solarbio) and LDH (Cat. No. BC0685; Solarbio) Activity Assay Kit according to the manufacturer's instructions, respectively. The SDH and LDH activities of the muscles were normalized to muscle weight, whereas the activity of myotubes was normalized to protein content.

### 2.15 Seahorse Analysis

A Mito Stress Test Kit (Cat. No. 103015‐100; Agilent Technologies, California, United States) was used to determine the oxygen consumption rate (OCR) according to the manufacturer's instructions. Briefly, C2C12 myoblasts were seeded in an XF96 cell culture microplate at a density of 1 × 10^4^ cells/well and fully differentiated. The cells were then incubated in an XF assay medium (Seahorse XF DMEM (pH 7.4) supplemented with 10‐mM glucose, 2‐mM pyruvate glutamine and 1‐mM pyruvate) and treated as indicated. The oligomycin, carbonyl cyanide‐4‐(trifluoromethoxy) phenylhydrazone (FCCP) and antimycin A/rotenone concentrations were 1.5, 1.5 and 0.5 μM, respectively. The OCR was determined and analysed using a Seahorse XF96 Analyser (Agilent Technologies).

### 2.16 Statistical Analysis

All data were presented as the mean ± SEM. Differences between groups were analysed using unpaired Student's *t*‐test or one‐way analysis of variance (ANOVA), followed by Tukey's test using GraphPad Prism 8 software. *p* < 0.05 was considered significant.

## 3 Results

### 3.1 MSC‐EXOs Alleviate Diabetes‐Induced Muscle Atrophy and Myofiber‐Type Transition

Here, we investigated the effects of hucMSC‐derived exosomes (MSC‐EXOs) on muscle atrophy and myofiber transitions in db/db mice. Flow cytometric analysis showed that hucMSCs were positive for CD105 and CD73 (> 95%) and negative for CD34 and HLA‐DR (< 2%) (Figure S1A). HucMSCs had the potential for adipogenic, osteogenic and chondrogenic differentiation, as characterized by Oil Red O, Alizarin Red S and Alcian Blue staining, respectively (Figure S1B–D). TEM (Figure S1E) and nanoparticle tracking analysis (NTA) (Figure S1F) suggested that MSC‐EXOs isolated from the hucMSC‐conditioned medium contained cup‐shaped vesicles with a diameter of ~130 nm. Western blot indicated that MSC‐EXOs were positive for the protein markers CD9, TSG101 and HSP70, whereas negative for calnexin (Figure S1G).

**FIGURE 1:**
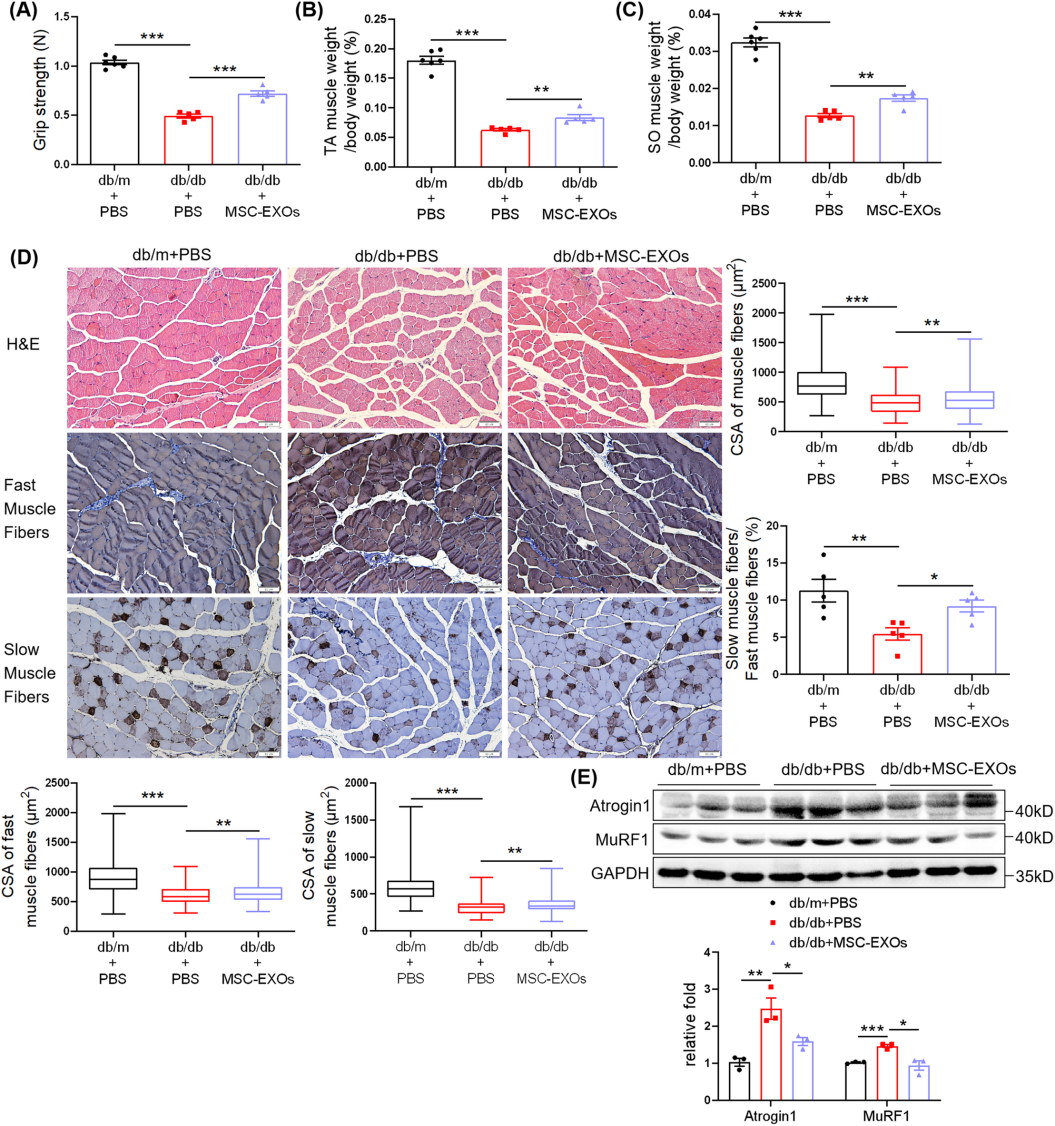
Mesenchymal stromal cell–derived exosomes (MSC‐EXOs) alleviate diabetes‐induced muscle atrophy and myofiber‐type transition. (A) Grip strength (*n* = 5–6 mice). (B) Percentage of tibialis anterior (TA) muscle weight in body weight (*n* = 5–6 mice). (C) Percentage of soleus (SO) muscle weight in body weight (*n* = 5–6 mice). (D) Haematoxylin–eosin (H&E) staining and immunohistochemical staining of fast and slow myosin heavy chain in TA muscles (scale bar, 50 μm). Cross‐sectional area (CSA) of muscle fibres, fast and slow muscle fibres, and the percentage of slow‐to‐fast muscle fibres (*n* = 5 mice). (E) Western blot analysis of Atrogin 1 and MuRF1 in TA muscles (*n* = 3 mice). Quantification of bands was performed using ImageJ software. Data are presented as mean ± SEM. (*p < 0.05, **p < 0.01 and ***p < 0.001).

IPGTT and IPITT indicated a decline in glucose and insulin tolerance in the db/db + PBS group compared to db/m + PBS group, which was elevated by MSC‐EXO injection (Figure S2A, B). The grip strength test suggested that MSC‐EXOs increased the muscle strength in db/db mice (Figure 1A). MSC‐EXOs injection did not affect body weight (Figure S2C) but elevated the TA and SO muscle mass (Figure 1B,C and S2D, E). H&E and immunohistochemical staining indicated lower levels of muscle fibre CSA in TA muscles, including fast and slow muscle fibres, and the percentage of slow‐to‐fast muscle fibres in db/db + PBS mice compared to db/m + PBS mice, which were all elevated by MSC‐EXO treatment (Figure 1D). Similar findings were observed for GAS muscles (Figure S3A–E). Additionally, MSC‐EXOs suppressed the diabetes‐associated upregulation of the E3‐ubiquitin ligases, Atrogin 1 and MuRF1 (Figure 1E). To further confirm the regulatory effect of MSC‐EXOs on the myofiber‐type transition, RT‐qPCR was performed to detect expression levels of muscle fibre type–related genes. Results showed that MSC‐EXOs upregulated messenger RNA (mRNA) levels of *MyHC I* (*Myh7*), *MyHC IIa* (*Myh7*), *Myoglobin*, *Tnni1* and *Tnnt1*, whereas they downregulated *MyHC IIb* (*Myh4*) expression (Figure S3F). These results indicated that MSC‐EXOs alleviated diabetes‐associated muscle atrophy and myofiber‐type transition in db/db mice.

### 3.2 MSC‐EXOs Rescue Atrophy‐Associated Impairment of the SIRT1/FoxO3a Signalling and Mitochondrial Function

**FIGURE 2:**
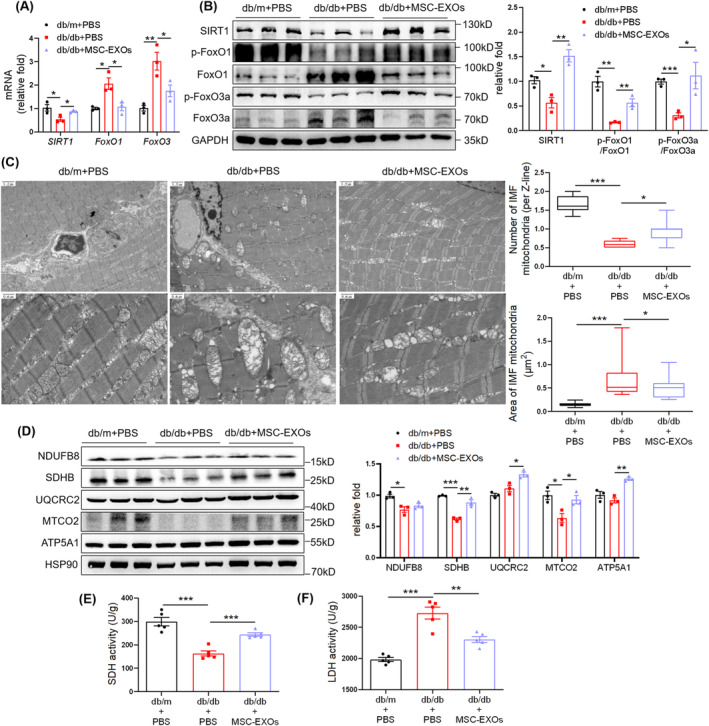
Mesenchymal stromal cell–derived exosomes (MSC‐EXOs) rescue atrophy‐associated impairment of the SIRT1/FoxO1/3a signalling and mitochondrial function. (A) RT‐qPCR analysis of *SIRT1*, *FoxO1* and *FoxO3* mRNA levels in TA muscles (*n* = 3 mice). (B) Western blot analysis of SIRT1, p‐FoxO1 (S319), FoxO1, p‐FoxO3a (S253) and FoxO3a in TA muscles (*n* = 3 mice). (C) Transmission electron microscopy (TEM) images of intermyofibrillar (IMF) mitochondria in TA muscles (scale bar, 1.5 μm/1.2 μm for above and 0.6 μm for below) and quantifications of IMF mitochondria number and size. (D) Western blot analysis of mitochondrial complex NDUFB8, SDHB, UQCRC2, MTCO2 and ATP5A1 in TA muscles of db/db mice (*n* = 3 mice). (E) SDH activity in gastrocnemius muscles of db/db mice (*n* = 5 mice). (F) LDH activity in gastrocnemius muscles of db/db mice (*n* = 5 mice). Quantification of bands was performed using ImageJ software. Data are presented as mean ± SEM. (*p < 0.05, **p < 0.01 and ***p < 0.001).

To explore the mechanism underlying MSC‐EXO‐mediated alleviation of muscle atrophy, we focused on the regulation of mitochondrial function and the related SIRT1/FoxO3a pathway. Lower *SIRT1* and higher *FoxO1 and FoxO3* mRNA levels were detected in the muscles of db/db + PBS mice than in db/m + PBS mice, while MSC‐EXO administration upregulated *SIRT1* and downregulated *FoxO1* and *FoxO3* mRNA levels (Figure 2A). Western blot suggested that MSC‐EXOs elevated the expression of SIRT1 and phosphorylation of FoxO1 and FoxO3a (Figure 2B). TEM indicated a decreased mitochondrial number, mitochondrial swelling and cristae fractures in muscles of db/db + PBS mice, whereas MSC‐EXO treatment upregulated mitochondrial number and rescued the diabetes‐induced impairment of mitochondrial structure (Figure 2C). The expression of mitochondrial complexes, including SDHB, UQCRC2, MTCO2 and ATP5A1, was elevated by MSC‐EXOs (Figure 2D). MSC‐EXOs also upregulated SDH activity but downregulated LDH activity (Figure 2E,F). These results demonstrate that MSC‐EXOs activate SIRT1/FoxO1/3a signalling pathways and alleviate mitochondrial dysfunction in skeletal muscles.

### 3.3 MSC‐EXOs Alleviate PA‐Induced C2C12 Myotube Atrophy and Mitochondrial Dysfunction

**FIGURE 3:**
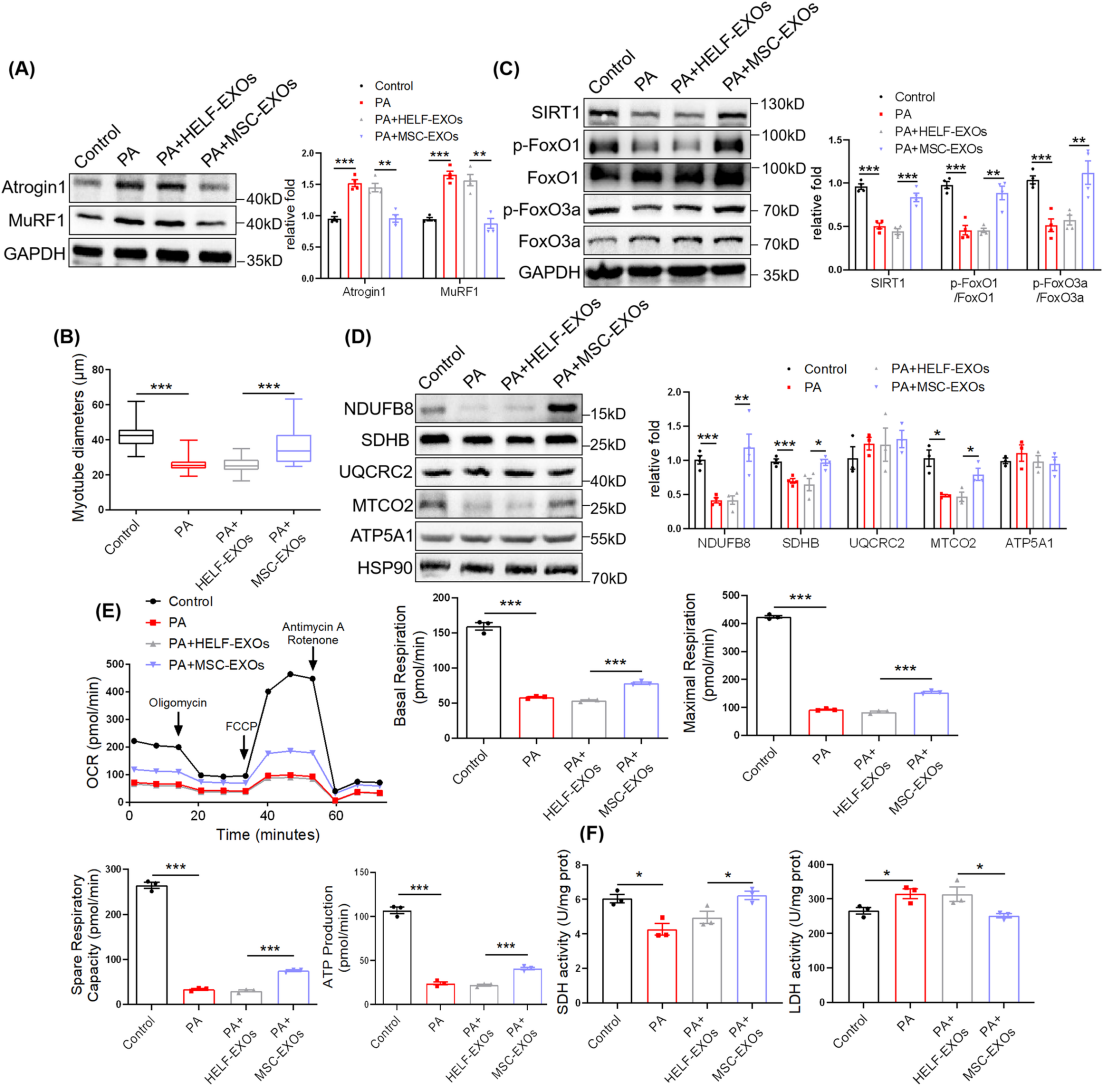
Mesenchymal stromal cell‐derived exosomes (MSC‐EXOs) alleviate PA‐induced C2C12 myotube atrophy and mitochondrial dysfunction. (A) Western blot analysis of Atrogin 1 and MuRF1 in C2C12 myotubes treated with PA and MSC‐EXOs (*n* = 4). (B) Diameters of C2C12 myotubes treated with PA and MSC‐EXOs. (C) Western blot analysis of SIRT1, p‐FoxO1 (S319), FoxO1, p‐FoxO3a (S253) and FoxO3a in C2C12 myotubes (*n* = 4). (D) Western blot analysis of mitochondrial complex NDUFB8, SDHB, UQCRC2, MTCO2 and ATP5A1 (*n* = 3–4). (E) Seahorse analysis of OXPHOS in C2C12 myotubes treated with PA and MSC‐EXO, including basal respiration, maximal respiration, spare respiratory capacity and ATP production (*n* = 3). (F) SDH and LDH activities in C2C12 myotubes treated with PA and MSC‐EXOs (*n* = 3). Quantification of bands was performed using ImageJ software. Data are presented as mean ± SEM. (*p < 0.05, **p < 0.01 and ***p < 0.001).

To further investigate the direct cell‐autonomous effect of MSC‐EXOs on the muscles, an in vitro C2C12 myocyte model was used. HELF‐EXOs were used as a control. PA treatment elevated the expression of Atrogin 1 and MuRF1 (Figure 3A) and reduced the diameter of myotubes (Figure 3B and S4A), whereas MSC‐EXOs treatment downregulated Atrogin 1 and MuRF1 expression and increased myotube diameters (Figure 3A,B and S4A). To explore the effect of MSC‐EXOs on myocyte differentiation, the expression of differentiation marker MyoD1 was detected and it was determined that MSC‐EXOs upregulated its protein level (Figure S4B). MSC‐EXOs increased SIRT1 expression and phosphorylation of FoxO1 and FoxO3a (Figure 3C) and upregulated the expression of mitochondrial complexes, including NDUFB8, SDHB and MTCO2 (Figure 3D). Seahorse analysis showed that MSC‐EXOs treatment upregulated mitochondrial OXPHOS, as evidenced by increased basal respiration, maximal respiration, spare respiratory capacity and ATP production (Figure 3E). In addition, MSC‐EXOs elevated SDH activity and reduced LDH activity in the myotubes (Figure 3F). These results support the direct cell‐autonomous effects of MSC‐EXOs on myocytes and suggest that MSC‐EXOs regulate SIRT1/FoxO1/3a signalling and mitochondrial function in vitro.

### 3.4 MSC‐EXOs Counteract Myotube Atrophy via Enhancing SIRT1‐Mediated Mitochondrial Function

**FIGURE 4:**
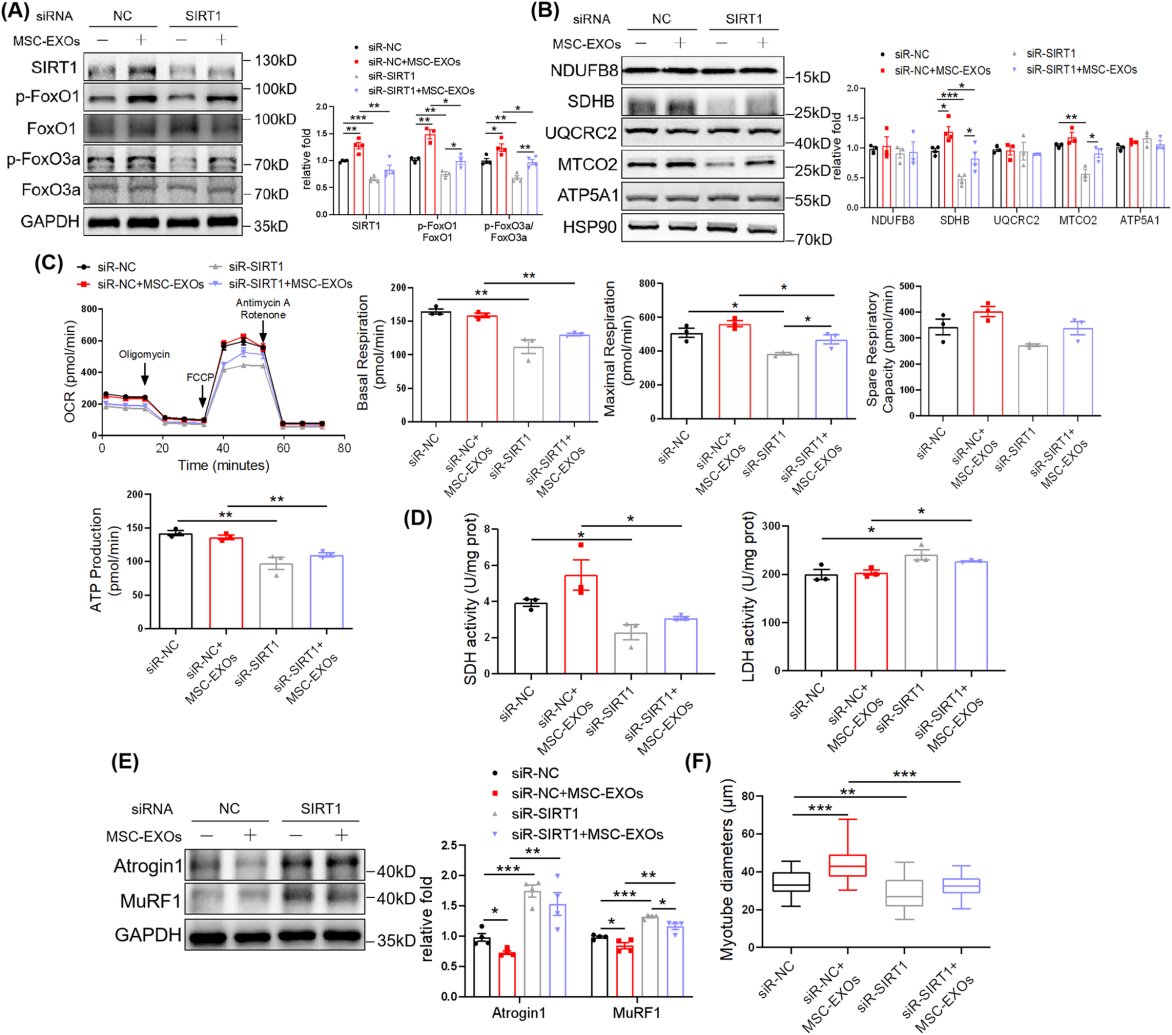
Mesenchymal stromal cell–derived exosomes (MSC‐EXOs) counteract myotube atrophy via enhancing SIRT1‐mediated mitochondrial function. (A) Western blot analysis of SIRT1, p‐FoxO1 (S319), FoxO1, p‐FoxO3a (S253) and FoxO3a in C2C12 myotubes transfected with SIRT1 siRNA and treated with MSC‐EXOs (*n* = 3–4). (B) Western blot analysis of mitochondrial complex NDUFB8, SDHB, UQCRC2, MTCO2 and ATP5A1 (*n* = 3–4). (C) Seahorse analysis of OXPHOS in C2C12 myotubes transfected with SIRT1 siRNA and treated with MSC‐EXOs, including basal respiration, maximal respiration, spare respiratory capacity and ATP production (*n* = 3). (D) SDH and LDH activities in C2C12 myotubes transfected with SIRT1 siRNA and treated with MSC‐EXOs (*n* = 3). (E) Western blot analysis of Atrogin 1 and MuRF1 (*n* = 4). (F) Diameters of C2C12 myotubes. Quantification of bands was performed using ImageJ software. Data are presented as mean ± SEM. (*p < 0.05, **p < 0.01 and ***p < 0.001).

To address whether SIRT1 is required for the effects of MSC‐EXOs on muscle atrophy, siRNA targeting SIRT1 was used to pretreat C2C12 myotubes. Western blot showed that MSC‐EXO mediated the upregulation of SIRT1/FoxO1/3a signalling, and mitochondrial complexes were partially abolished by siR‐SIRT1 (Figure 4A,B). Simultaneously, SIRT1 knockdown diminished the effects of MSC‐EXOs on OXPHOS (Figure 4C), SDH and LDH activity (Figure 4D) in myotubes. Consequently, siR‐SIRT1 weakened the MSC‐EXO‐dependent decrease in Atrogin 1/MuRF1 levels (Figure 4E) and increase in myotube diameters (Figure 4F and S5A). To further confirm the role of SIRT1 under the effect of MSC‐EXOs, C2C12 myoblasts were also transfected with shRNA via lentivirus before being fully differentiated. It was determined that SIRT1 expression could be efficiently decreased by shRNA. sh‐SIRT1 mimicked siR‐SIRT1 in diminishing MSC‐EXO effects in SIRT1/FoxO1/3a signalling, mitochondrial complexes, Atrogin 1/MuRF1 levels and myotube diameters (Figure S5B–E). These results indicated that MSC‐EXOs alleviated muscle atrophy by promoting SIRT1‐mediated mitochondrial function in C2C12 myotubes.

### 3.5 Apt Conjugation Facilitates the Internalisation Efficacy of MSC‐EXOs in Skeletal Muscles

**FIGURE 5:**
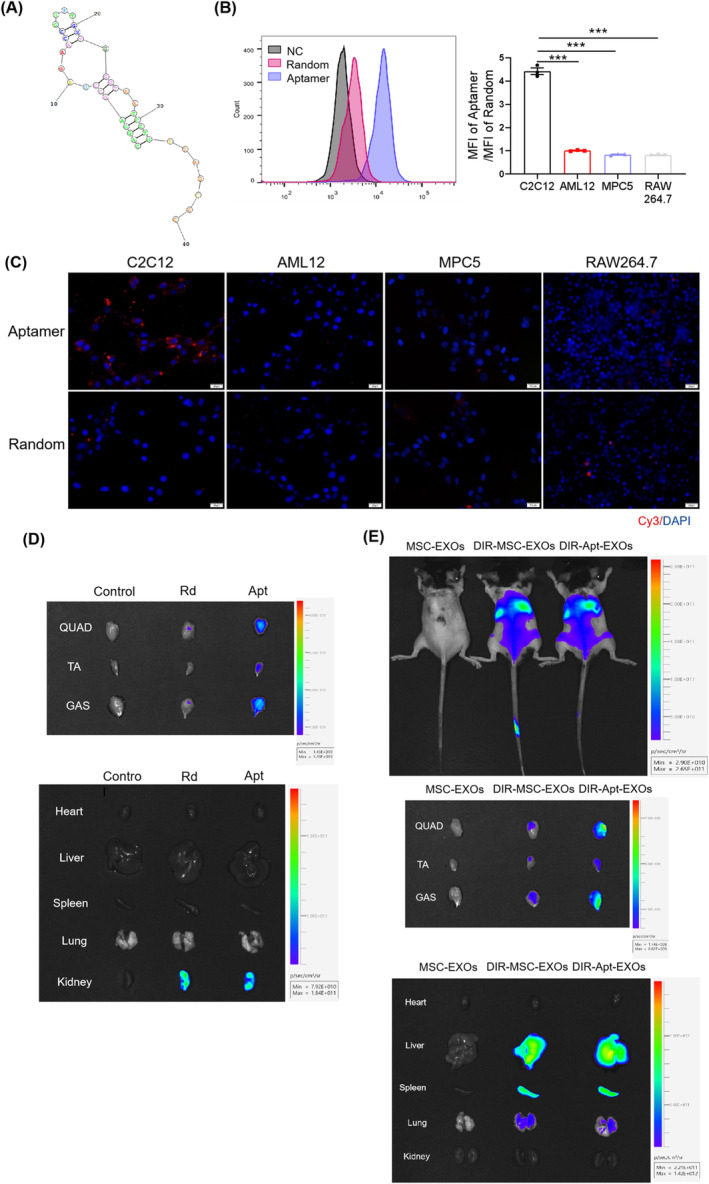
The Aptamer (Apt) conjugation facilitates the internalisation efficacy of mesenchymal stromal cell–derived exosomes (MSC‐EXOs) in skeletal muscles. (A) Predicted secondary structure of the Apt. (B) Affinity of the Apts for myocytes by flow cytometry analysis (*n* = 3). (C) Affinity of the Apts for myocytes captured by fluorescence microscopy. (D) Distribution of Cy3‐labelled Apts in mice after 30 min of infusion by an in vivo imaging system (IVIS), including quadriceps (QUAD), tibialis anterior (TA), gastrocnemius muscles (GAS), heart, liver, spleen, lung and kidney. (E) Distribution of DIR‐labelled Apt‐conjugated MSC‐EXOs in mice after 6 h of infusion by an IVIS, including QUAD, TA, GAS, heart, liver, spleen, lung and kidney. Data are presented as mean ± SEM. (***p < 0.001).

Next, we hypothesised that modification of MSC‐EXOs with specific recognisable ligands would achieve muscle‐targeted delivery and enhance the effects of MSC‐EXOs on muscle atrophy. The RNAstructure website was used to predict the secondary structure of the Apts (Figure 5A). To confirm their specificity, the Apts were labelled with Cy3 and incubated with various cell types, including myoblasts C2C12, hepatocytes AML12, podocytes MPC5 and macrophages/monocytes RAW264.7, as reported previously [24]. Flow cytometry analysis showed that the Apts had a greater affinity for myocytes than for other cell types (*p* < 0.05; Figure 5B). The picture obtained via fluorescence microscopy revealed that Cy3‐labelled Apts specifically entered C2C12 myoblasts but could not enter AML12, MPC5 and RAW264.7 cells (Figure 5C). Meanwhile, in vivo tracing indicated that the Apts accumulated specifically in the skeletal muscles (Figure 5D).

To deliver EXOs to skeletal muscles, the above‐mentioned Apts were conjugated to MSC‐EXOs. 5′‐end of the Apts were modified with an aldehyde group, which could react with an amino group on the membrane of MSC‐EXOs via the Schiff base reaction (Figure S6A). TEM, NTA and western blot analyses showed that Apt conjugation did not influence the fundamental characterisation of MSC‐EXOs (Figure S6B–D) but promoted the internalisation efficacy of MSC‐EXOs in myocytes (Figure S6E). Notably, in vivo biodistribution of the systemically delivered Apt conjugate, MSC‐EXOs, was detected. IVIS analysis suggested that apt conjugation facilitated the internalisation of MSC‐EXOs into skeletal muscles (Figure 5E).

### 3.6 Apt Conjugation Strengthens the Effects of MSC‐EXOs on Muscle Atrophy and Myofiber‐Type Transition

**FIGURE 6:**
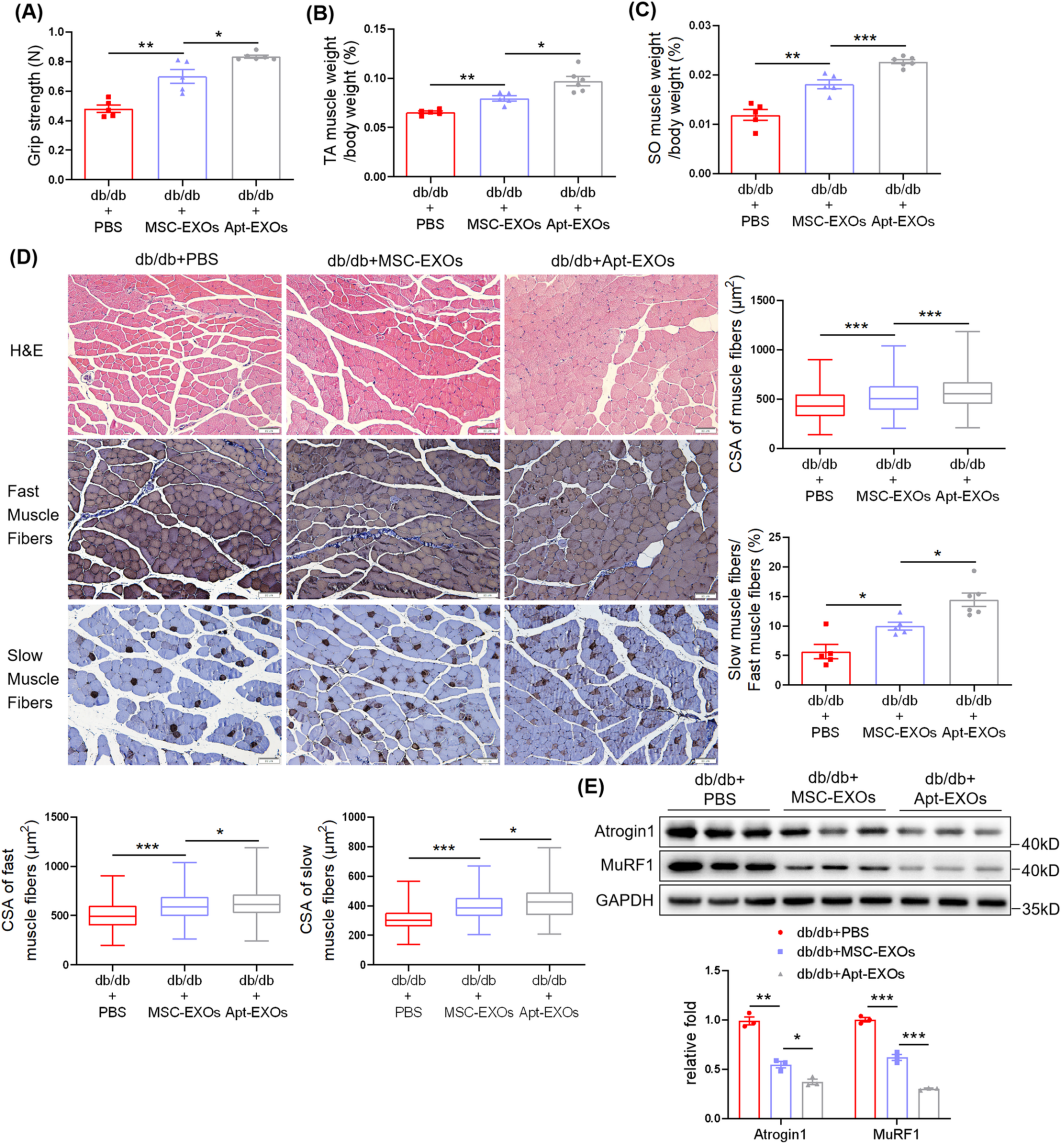
Aptamer (Apt) conjugation strengthens the effects of mesenchymal stromal cell–derived exosomes (MSC‐EXOs) on muscle atrophy and myofiber‐type transition. (A) Grip strength (*n* = 5–6 mice). (B) Percentage of tibialis anterior (TA) muscle weight in body weight (*n* = 5–6 mice). (C) Percentage of soleus (SO) muscle weight in body weight (*n* = 5–6 mice). (D) H&E staining and immunohistochemical staining of fast and slow myosin heavy chain in TA muscles (scale bar, 50 μm). Cross‐sectional area (CSA) of muscle fibres, fast and slow muscle fibres and the percentage of slow‐to‐fast muscle fibres (*n* = 5–6 mice). (E) Western blot analysis of Atrogin 1 and MuRF1 in TA muscles (*n* = 3 mice). Quantification of bands was performed using ImageJ software. Data are presented as mean ± SEM. (*p < 0.05, **p < 0.01 and ***p < 0.001).

The IPGTT and IPITT showed no significant differences between the db/db + Apt‐EXOs and db/db + MSC‐EXOs groups (Figure S7A, B). However, Apt‐EXO treatment further enhanced the grip strength of db/db mice (Figure 6A) and increased TA and SO muscle mass without affecting body weight (Figure 6B,C and S7C–E) compared with MSC‐EXOs. H&E and immunohistochemical staining showed that Apt‐EXOs further increased the CSA of muscle fibres, including fast and slow muscle fibres; increased the percentage of slow muscle fibres (Figure 6D); and inhibited Atrogin 1/MuRF1 expression (Figure 6E). These results demonstrate that Apt conjugation strengthens the effects of MSC‐EXOs on muscle atrophy and myofiber transition.

### 3.7 Apt Conjugation Enhances the Effects of MSC‐EXOs via SIRT1‐Mediated Mitochondrial Function

**FIGURE 7:**
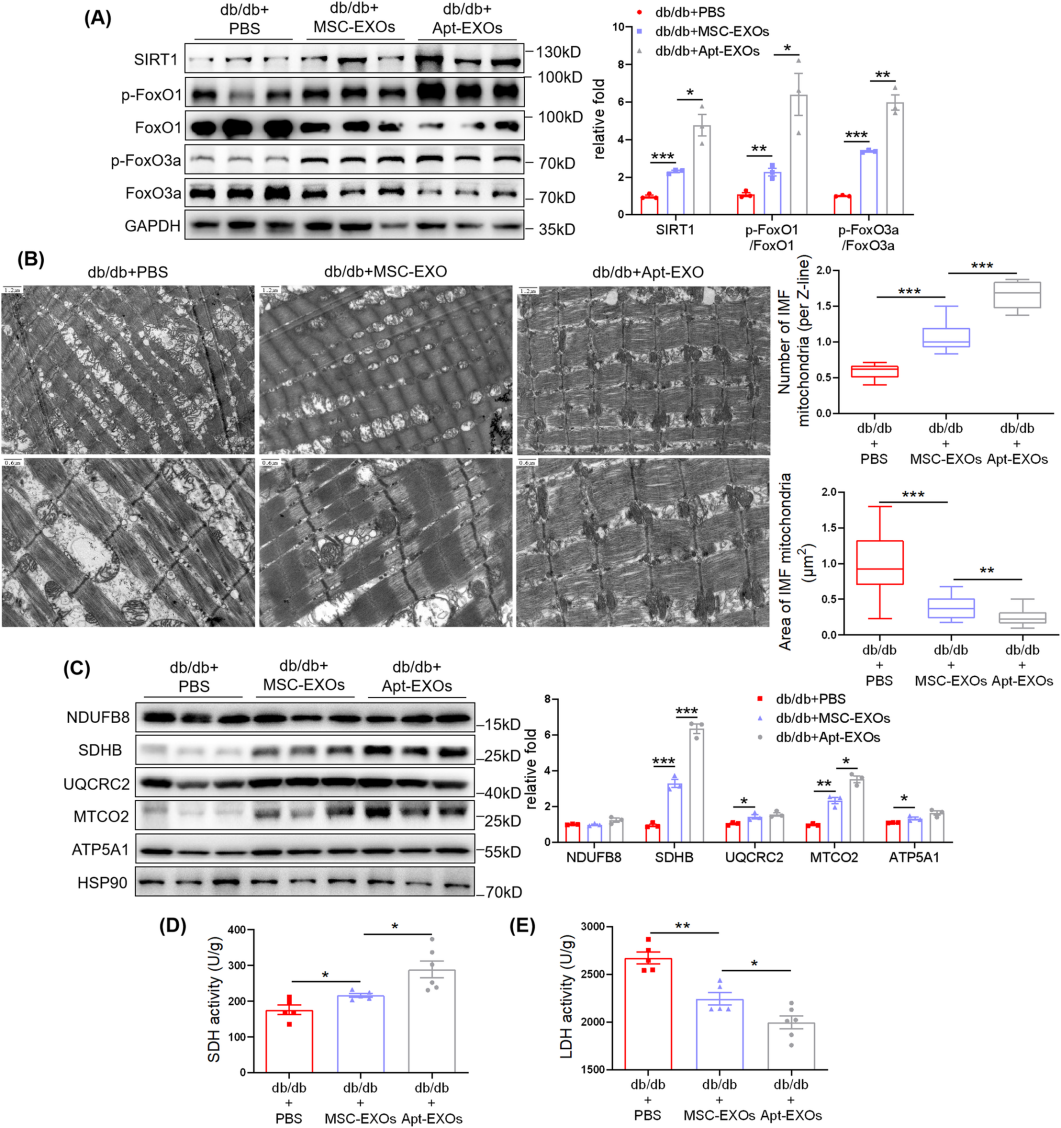
Aptamer (Apt) conjugation enhances the effects of mesenchymal stromal cell–derived exosomes (MSC‐EXOs) via SIRT1‐mediated mitochondrial function. (A) Western blot analysis of SIRT1, p‐FoxO1 (S319), FoxO1, p‐FoxO3a (S253) and FoxO3a in TA muscles of db/db mice (*n* = 3 mice). (B) TEM images of IMF mitochondria in TA muscles (scale bar, 1.5 μm/1.2 μm for above and 0.6 μm for below) and quantifications of IMF mitochondria number and size. (C) Western blot analysis of mitochondrial complex NDUFB8, SDHB, UQCRC2, MTCO2 and ATP5A1 in TA muscles (*n* = 3 mice). (D) SDH activity in gastrocnemius muscles (*n* = 5–6 mice). (E) LDH activity in gastrocnemius muscles (*n* = 5–6 mice). Quantification of bands was performed using ImageJ software. Data are presented as mean ± SEM. (*p < 0.05, **p < 0.01 and ***p < 0.001).

To further verify the regulatory mechanism of Apt‐EXOs in muscle atrophy, we examined SIRT1 signalling and mitochondrial function. Western blot showed that the expression of SIRT1 and phosphorylation of FoxO1 and FoxO3a were higher in the db/db + Apt‐EXOs group than that in the db/db + MSC‐EXOs group (Figure 7A). TEM indicated that the mitochondrial number and structure in the db/db + Apt‐EXO group were better than those in the db/db + MSC‐EXOs group (Figure 7B). The expression of mitochondrial complexes, including SDHB and MTCO2, was further elevated by Apt‐EXOs (Figure 7C). Additionally, Apt‐EXOs enhanced SDH activity and reduced LDH activity (Figure 7D,E). These results indicate that Apt conjugation might enhance the effects of MSC‐EXOs via SIRT1‐mediated mitochondrial function.

## 4 Discussion

Skeletal muscle atrophy reduces quality of life and increases mortality in patients with T2DM [2, 3]. However, no specific strategies are currently available. MSC‐based therapy has proven effective in alleviating muscle atrophy caused by various factors [25, 26]. It was originally thought that administered MSCs could migrate to the sites of injury, implant and differentiate into functional cells, leading to the repair of damaged or diseased connective tissues. However, in vivo tracing showed that MSCs were short‐lived following intravenous infusion. Meanwhile, most MSCs are located in the lungs, and only a small portion can enter the liver, spleen and damaged tissues [27]. Accumulating evidence suggests that MSCs exert therapeutic effects through paracrine action [16] (S3). Exosomes secreted by MSCs are extracellular vesicles with a diameter between 30 and 200 nm that contain a variety of bioactive molecules such as mRNA, microRNA (miRNA), cytokines, chemokines and immunomodulators, which play an important role in paracrine [17]. Compared to MSCs, EXOs are more readily available and have lower immunogenicity and tumorigenicity in vivo, which makes them ideal for regenerative therapy [19] (S4, S5). Moreover, multiple basic and clinical studies have confirmed the safety of MSC‐EXOs [28] (S7, S8). In the current study, in vivo tracing showed that DIR‐labelled MSC‐EXOs could successfully reach the skeletal muscles of mice. MSC‐EXO treatment increased muscle strength and mass in db/db mice. Meanwhile, MSC‐EXOs ameliorate PA‐induced muscle atrophy in C2C12 myotubes.

However, systematical application of EXOs has lower efficacy and safety due to their poor selectivity for diseased tissues in vivo. Improving EXO targeting enhances their bioavailability and tolerability, which is promising in regenerative therapy [20]. Currently, more and more researchers focus on SELEX technology to screen cell or tissue‐specific Apts, which form three‐dimensional structures and binding target molecules with high affinity and specificity. It has been used to identify diseased tissues in a covalent or physical coupling manner [21] (S6). Sun et al. selected and identified the human skeletal muscle–specific ssDNA Apts for the first time, which preferentially interacted with human skeletal muscle cells in vitro and in vivo [29]. Philippou et al. identified a skeletal muscle–specific RNA Apt using C2C12 myoblasts, which preferentially internalises in skeletal muscle cells and exhibits decreased affinity for off‐target cells. Moreover, this in vitro selected Apts retained its function in vivo [24]. The selective targeting properties of the Apts prompted us to investigate whether the conjugation of EXOs to muscle‐specific Apts could promote the preferential recruitment of EXOs to skeletal muscles and enhance their efficacy in muscle atrophy. Here, we used a previously reported muscle‐specific RNA Apt [24] and found that the Apt had a greater affinity for myocytes than other cell types. Meanwhile, in vivo tracing showed that the Apt accumulated specifically in skeletal muscles. Subsequently, the above Apt was conjugated to MSC‐EXOs, which facilitated the internalisation efficacy of MSC‐EXOs in skeletal muscles. This suggests that Apts may play a role by promoting EXO recruitment to target tissues, which is also reported previously [23]. Subsequently, we injected Apt‐ or MSC‐EXOs into db/db mice via the tail vein and demonstrated that Apt conjugation strengthened the effects of MSC‐EXOs on diabetes‐induced muscle atrophy, providing a new idea to improve the efficacy of EXOs in the treatment of muscle diseases.

Skeletal muscles are mainly composed of slow‐twitch oxidative (Type I) fibres and fast‐twitch glycolytic (Type II) fibres. Slow‐oxidative fibres have a higher number of mitochondria and exhibit higher OXPHOS capacity, which is closely related to exercise endurance [4, 5]. The myofiber‐type transition is accompanied by the change of mitochondrial content and function, which will affect the oxidative metabolism and muscle strength [3]. Studies have shown that mitochondrial dysfunction contributes to muscle atrophy [7, 8]. Disorders of glucose and lipid metabolism, insulin resistance and inflammatory responses in diabetes lead to mitochondrial dysfunction in skeletal muscles, including abnormal mitochondrial morphology, reduced mitochondrial capacity and upregulated expression of genes related to oxidative metabolism, which accelerate muscle atrophy [9, 10] (S1). Our results showed that in db/db mice, the mitochondrial structure of skeletal muscles was obviously impaired, accompanied by a decreased percentage of slow‐to‐fast muscle fibres. However, MSC‐EXO treatment rescued the abnormal mitochondrial structure, upregulated the expression of the mitochondrial complex, enhanced SDH activity and reduced LDH activity. What's more, MSC‐EXOs promoted the conversion of muscle fibre type from fast glycolytic to slow oxidative, which we considered an important reason for the increase in muscle strength. Seahorse analysis directly showed that MSC‐EXOs promoted mitochondrial OXPHOS in C2C12 myotubes, as evidenced by increased basal respiration, maximal respiration, spare respiratory capacity and ATP production. As reported previously, MSC‐derived extracellular vesicles or EXOs exert a protective effect on target organs by enhancing mitochondrial function [30, 31]. Additionally, aptamer‐conjugated MSC‐EXOs restored the mitochondrial structure and function in db/db mice. This study firstly reveals the mechanism by which MSC‐EXOs regulate muscle atrophy from the perspective of mitochondrial function.

FoxO transcription factors are critical in muscle atrophy, among which FoxO3 dephosphorylation directly activates muscle‐specific ubiquitin ligases and autophagy lysosomes [32]. Active FoxOs act on the Atrogin 1 promoter to cause its transcription and dramatic atrophy of myotubes and muscle fibres [33]. Sirtuins, particularly SIRT1, are associated with metabolic diseases such as obesity and diabetes. SIRT1 overexpression reduces muscle wasting by blocking the activation of FoxO1 and FoxO3 [14], and SIRT1 activation increased FoxO3a phosphorylation and decreased FoxO3a acetylation, which, in turn, potentially prevented the nuclear translocation of FoxO3a and suppressed the expression of Atrogin 1 and MuRF1 [S9]. Meanwhile, SIRT1 activation in the skeletal muscles of diabetic mice promotes mitochondrial biosynthesis [15]. It is reported that MSC‐derived extracellular vesicles or EXOs improve various diseases via upregulating SIRT1 signalling, such as spinal cord injury, lung injury and aging‐related vascular diseases [34, 35, 36], whereas the effect of MSC‐EXOs on SIRT1 expression in skeletal muscle remained unclear. In the present study, MSC‐EXOs promoted SIRT1/FoxO1/3a signal transduction in the skeletal muscles of db/db mice and in PA‐treated C2C12 myotubes. SIRT1 knockdown diminished the effects of MSC‐EXOs on OXPHOS, SDH and LDH activity and myotube diameter. These results indicated that SIRT1 participates in the amelioration of mitochondrial dysfunction by MSC‐EXOs, providing a new scientific basis and intervention target for EXOs to treat muscle atrophy. However, the potential key biological molecules in MSC‐EXOs that were responsible for regulating SIRT1 pathway to improve muscle atrophy have not been explored. It is reported that there is abundant calcium/calmodulin‐dependent protein kinase (CAMKK) which mediates the effect of MSC‐EXOs [37]. CAMKK plays a protective role in the endothelium and lung tissue by activating SIRT1 [38, 39]. Further investigation whether CAMKK is a key molecule secreted by MSC‐EXOs in the regulation of SIRT1 involved in muscle atrophy is needed.

In conclusion, this study first reveals the mechanism by which MSC‐EXOs regulate muscle atrophy from the perspective of mitochondrial function. Moreover, the results suggest that hucMSC‐secreted EXOs enhance SIRT1/FoxO1/3a‐mediated mitochondrial function and ameliorate muscle atrophy in diabetes. Apt conjugation strengthens the effects of MSC‐EXOs on muscle atrophy. These findings demonstrate the therapeutic potential of muscle‐targeted MSC‐EXOs for the treatment of muscle atrophy.

## Ethics Statement

The authors certify that they complied with the ethical guidelines for authorship and publication in the *Journal of Cachexia, Sarcopenia and Muscle* [40]. All animal studies were approved by the Animal Ethics Committee of Shandong University and were performed in accordance with the ethical standards of the 1964 Declaration of Helsinki and its later amendments. The manuscript does not contain any clinical studies or patient data.

## Consent

The authors have nothing to report.

## Conflicts of Interest

The authors declare no conflicts of interest.

## Supporting information


**Fig. S1** Identification of human umbilical cord mesenchymal stromal cells (hucMSCs) and mesenchymal stromal cell–derived exosomes (MSC‐EXOs). (A) Flow cytometry analysis of hucMSCs markers CD105, CD73, CD34 and HLA‐DR. (B) Oil Red O staining for adipogenic differentiation ability of hucMSCs (scale bar, 20 μm). (C) Alizarin Red S staining for osteogenic differentiation ability of hucMSCs (scale bar, 20 μm). (D) Alcian Blue staining for chondrogenic differentiation ability of hucMSCs (scale bar, 100 μm). (E) TEM images of MSC‐EXOs. (F) Nanoparticle tracking analysis of exosomal sizes. (G) Western blot analysis of the exosomal markers CD9, TSG101, HSP70 and endoplasmic reticulum marker calnexin of MSC‐EXOs.
**Figure S2.** MSC‐EXOs alleviate diabetes‐induced muscle atrophy. (A) Intraperitoneal glucose tolerance test (IPGTT) and area under the curve (AUC) of db/db mice after MSC‐EXO injection (*n* = 5–6 mice; *db/db + PBS group vs. db/m + PBS group; ^#^db/db + MSC‐EXOs group vs db/db + PBS group). (B) Intraperitoneal insulin tolerance test (IPITT) and AUC of db/db mice after MSC‐EXO injection (*n* = 5–6 mice). (C) Body weight (*n* = 5–6 mice). (D) Tibialis anterior (TA) muscle weight (*n* = 5–6 mice). (E) Soleus (SO) muscle weight (*n* = 5–6 mice). Data are mean ± SEM. (*p < 0.05, **p < 0.01 and ***p < 0.001).
**Figure S3.** MSC‐EXOs alleviate diabetes‐induced muscle atrophy and myofiber‐type transition. (A) H&E and immunohistochemical staining of fast and slow myosin heavy chain in GAS muscles (scale bar, 50 μm). (B) Cross‐sectional area (CSA) of muscle fibres (*n* = 4–5 mice). (C) CSA of fast muscle fibres (*n* = 4–5 mice). (D) CSA of slow muscle fibres (*n* = 4–5 mice). (E) The percentage of slow to fast muscle fibres (*n* = 4–5 mice). (F) RT‐qPCR analysis of *MyHC I* (*Myh7*), *MyHC IIa* (*Myh7*), *MyHC IIb* (*Myh4*), *Myoglobin*, *Tnni1* and *Tnnt1* mRNA levels in TA muscles (*n* = 5 mice). Data are mean ± SEM. (*p < 0.05, **p < 0.01 and ***p < 0.001).
**Figure S4.** MSC‐EXOs alleviate PA‐induced C2C12 myotube atrophy. (A) Imaging of C2C12 myotubes treated with PA and MSC‐EXOs (scale bar, 50 μm). (B) Western blot analysis of differentiation marker MyoD1 (*n* = 4). Quantification of bands was performed using ImageJ software. Data are mean ± SEM. (**p < 0.01 and ***p < 0.001).
**Figure S5.** MSC‐EXOs counteracts myotube atrophy via enhancing SIRT1‐mediated mitochondrial function. (A) Imaging of C2C12 myotubes transfected with SIRT1 siRNA and treated with MSC‐EXOs (scale bar, 50 μm). (B) Western blot analysis of SIRT1, p‐FoxO1 (S319), FoxO1, p‐FoxO3a (S253) and FoxO3a in C2C12 myotubes transfected with SIRT1 shRNA and treated with MSC‐EXOs (*n* = 3–4). (C) Western blot analysis of mitochondrial complex NDUFB8, SDHB, UQCRC2, MTCO2 and ATP5A1 (*n* = 3–4). (D) Western blot analysis of Atrogin 1 and MuRF1 (*n* = 3–4). (E) Imaging of C2C12 myotubes transfected with SIRT1 shRNA and treated with MSC‐EXOs (scale bar, 50 μm). (F) Diameters of C2C12 myotubes. Quantification of bands was performed using ImageJ software. Data are mean ± SEM. (*p < 0.05, **p < 0.01 and ***p < 0.001).
**Figure S6.** The effects of aptamer conjugation on MSC‐EXOs. (A) *C*onjugation of aptamer to MSC‐EXOs via the Schiff base reaction. (B) TEM images of aptamer‐functionalized exosomes (Apt‐EXOs). (C) Western blot analysis of the exosomal markers CD9, TSG101, HSP70 and endoplasmic reticulum marker calnexin of Apt‐EXOs. (D) Nanoparticle tracking analysis of exosomal sizes. (E) Fluorescence‐tracing of PKH67‐labelled MSC‐EXO/Apt‐EXO uptake by C2C12 myoblasts (scale bar, 20 μm).
**Figure S7.** Aptamer conjugation strengthens the effects of MSC‐EXOs on muscle atrophy. (A) IPGTT and AUC of db/db mice after Apt‐EXO injection (*n* = 5–6 mice). (B) IPITT and AUC of db/db mice after Apt‐EXO injection (*n* = 5–6 mice). (C) Body weight (*n* = 5–6 mice). (D) TA muscle weight (*n* = 5–6 mice). (E) SO muscle weight (*n* = 5–6 mice). Data are mean ± SEM. (*p < 0.05, **p < 0.01 and ***p < 0.001).

**Data S1** Supplementary Information.

## Data Availability

All data generated or analysed during this study are included in this published article.
