# Supplementary material for: Aptamer‐Conjugated Exosomes Ameliorate Diabetes‐Induced Muscle Atrophy by Enhancing SIRT1/FoxO1/3a‐Mediated Mitochondrial Function
Source: J Cachexia Sarcopenia Muscle. 2025 Jan 28;16(1):e13717. doi: 10.1002/jcsm.13717 (PMC11773161; doi:10.1002/jcsm.13717)
Supplement: Supplementary file 1 — Fig. S1 Identification of human umbilical cord mesenchymal stromal cells (hucMSCs) and mesenchymal stromal cell–derived exosomes (MSC‐EXOs). (A) Flow cytometry analysis of hucMSCs markers CD105, CD73, CD34 and HLA‐DR. (B) Oil Red O staining for adipogenic differentiation ability of hucMSCs (scale bar, 20 μm). (C) Alizarin Red S staining for osteogenic differentiation ability of hucMSCs (scale bar, 20 μm). (D) Alcian Blue staining for chondrogenic differentiation ability of hucMSCs (scale bar, 100 μm). (E) TEM images of MSC‐EXOs. (F) Nanoparticle tracking analysis of exosomal sizes. (G) Western blot analysis of the exosomal markers CD9, TSG101, HSP70 and endoplasmic reticulum marker calnexin of MSC‐EXOs. Figure S2. MSC‐EXOs alleviate diabetes‐induced muscle atrophy. (A) Intraperitoneal glucose tolerance test (IPGTT) and area under the curve (AUC) of db/db mice after MSC‐EXO injection (n = 5–6 mice; *db/db + PBS group vs. db/m + PBS group; #db/db + MSC‐EXOs group vs db/db + PBS group). (B) Intraperitoneal insulin tolerance test (IPITT) and AUC of db/db mice after MSC‐EXO injection (n = 5–6 mice). (C) Body weight (n = 5–6 mice). (D) Tibialis anterior (TA) muscle weight (n = 5–6 mice). (E) Soleus (SO) muscle weight (n = 5–6 mice). Data are mean ± SEM. (*p < 0.05, **p < 0.01 and ***p < 0.001). Figure S3. MSC‐EXOs alleviate diabetes‐induced muscle atrophy and myofiber‐type transition. (A) H&E and immunohistochemical staining of fast and slow myosin heavy chain in GAS muscles (scale bar, 50 μm). (B) Cross‐sectional area (CSA) of muscle fibres (n = 4–5 mice). (C) CSA of fast muscle fibres (n = 4–5 mice). (D) CSA of slow muscle fibres (n = 4–5 mice). (E) The percentage of slow to fast muscle fibres (n = 4–5 mice). (F) RT‐qPCR analysis of MyHC I (Myh7), MyHC IIa (Myh7), MyHC IIb (Myh4), Myoglobin, Tnni1 and Tnnt1 mRNA levels in TA muscles (n = 5 mice). Data are mean ± SEM. (*p < 0.05, **p < 0.01 and ***p < 0.001). Figure S4. MSC‐EXOs alleviate PA‐induced C2C12 myotube atrophy [file JCSM-16-e13717-s001.docx]

**
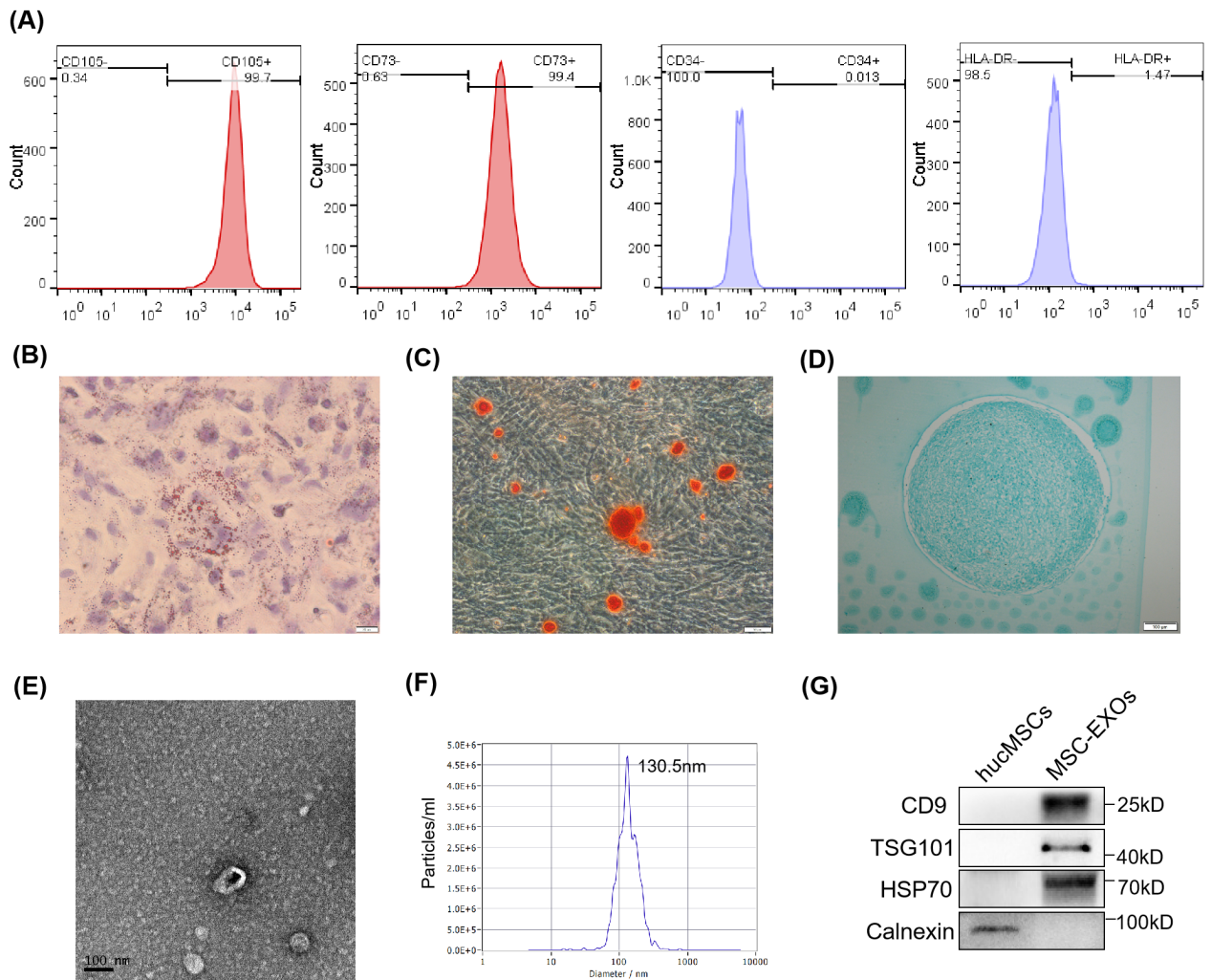
Supplementary Figures**

**Supplementary Fig. 1** Identification of human umbilical cord mesenchymal stromal cells (hucMSCs) and mesenchymal stromal cell-derived exosomes (MSC-EXOs). *(A)* Flow cytometry analysis of hucMSCs markers CD105, CD73, CD34, and HLA-DR. *(B)* Oil Red O staining for adipogenic differentiation ability of hucMSCs (Scale bar, 20 μm). *(C)* Alizarin Red S staining for osteogenic differentiation ability of hucMSCs (Scale bar, 20 μm). *(D)* Alcian Blue staining for chondrogenic differentiation ability of hucMSCs (Scale bar, 100 μm). *(E)* TEM images of MSC-EXOs. *(F)* Nanoparticle tracking analysis of exosomal sizes. *(G)* Western blot analysis of the exosomal markers CD9, TSG101, HSP70, and endoplasmic reticulum marker Calnexin of MSC-EXOs.


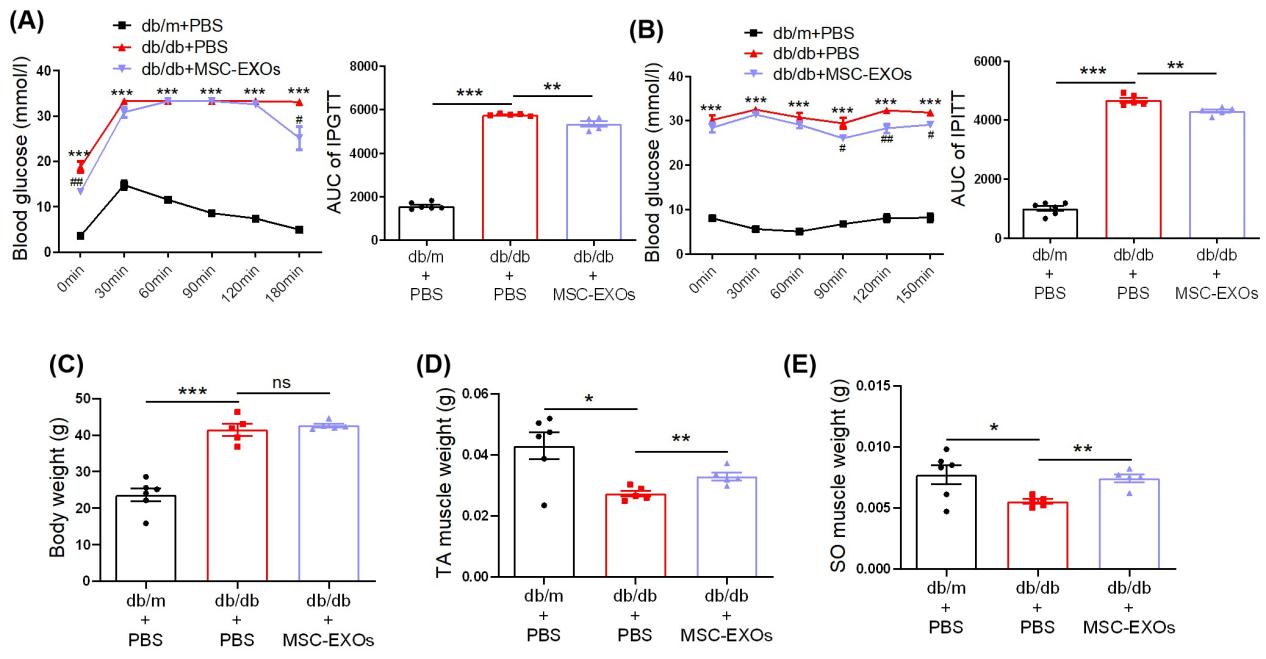


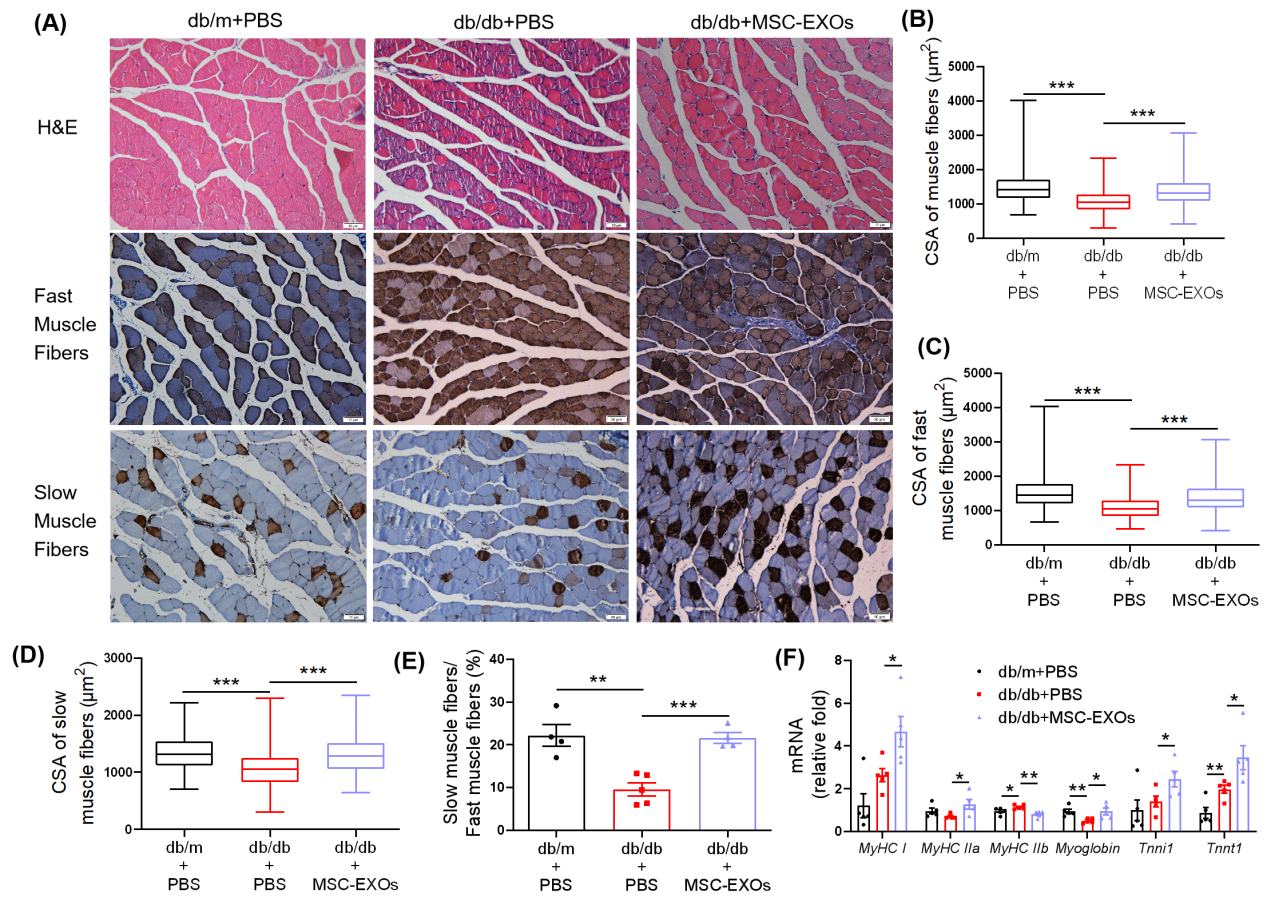
**Supplementary Fig. 2** MSC-EXOs alleviate diabetes-induced muscle atrophy. *(A)* Intraperitoneal glucose tolerance test (IPGTT) and area under the curve (AUC) of db/db mice after MSC-EXO injection (n = 5–6 mice. *db/db+PBS group vs. db/m+PBS group; ^#^ db/db+MSC-EXOs group vs db/db+PBS group). *(B)* Intraperitoneal insulin tolerance test (IPITT) and AUC of db/db mice after MSC-EXO injection (n = 5–6 mice). *(C)* Body weight (n = 5–6 mice). *(D)* Tibialis anterior (TA) muscle weight (n = 5–6 mice). *(E)* Soleus (SO) muscle weight (n = 5–6 mice). Data are mean ± SEM. (**P* < 0.05, ***P* < 0.01, ****P* < 0.001)

**Supplementary Fig. 3** MSC-EXOs alleviate diabetes-induced muscle atrophy and myofiber-type transition. *(A)* H&E and immunohistochemical staining of fast and slow myosin heavy chain in GAS muscles (Scale bar, 50 μm). *(B)* Cross-sectional area (CSA) of muscle fibers (n = 4-5 mice). *(C)* CSA of fast muscle fibers (n = 4-5 mice). *(D)* CSA of slow muscle fibers (n = 4-5 mice). *(E)* The percentage of slow to fast muscle fibers (n = 4-5 mice). *(F)* RT-qPCR analysis of *MyHC I(Myh7), MyHC IIa(Myh7), MyHC IIb(Myh4), Myoglobin, Tnni1* and *Tnnt1* mRNA levels in TA muscles (n = 5 mice). Data are mean ± SEM. (**P* < 0.05, ***P* < 0.01, ****P* < 0.001)


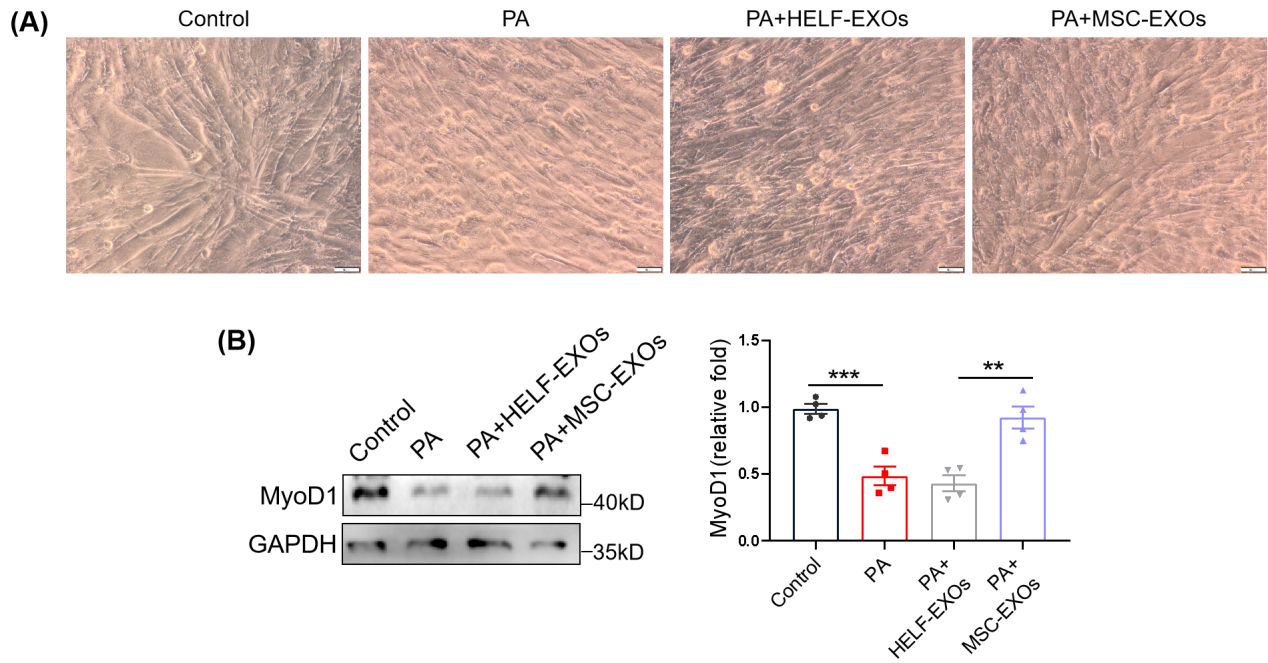


**Supplementary Fig. 4** MSC-EXOs alleviate PA-induced C2C12 myotube atrophy. *(A)* Imaging of C2C12 myotubes treated with PA and MSC-EXOs (Scale bar, 50 μm). *(B)* Western blot analysis of differentiation marker MyoD1 (n=4). Quantification of bands was performed using ImageJ software. Data are mean ± SEM. (***P* < 0.01, ****P* < 0.001)

**
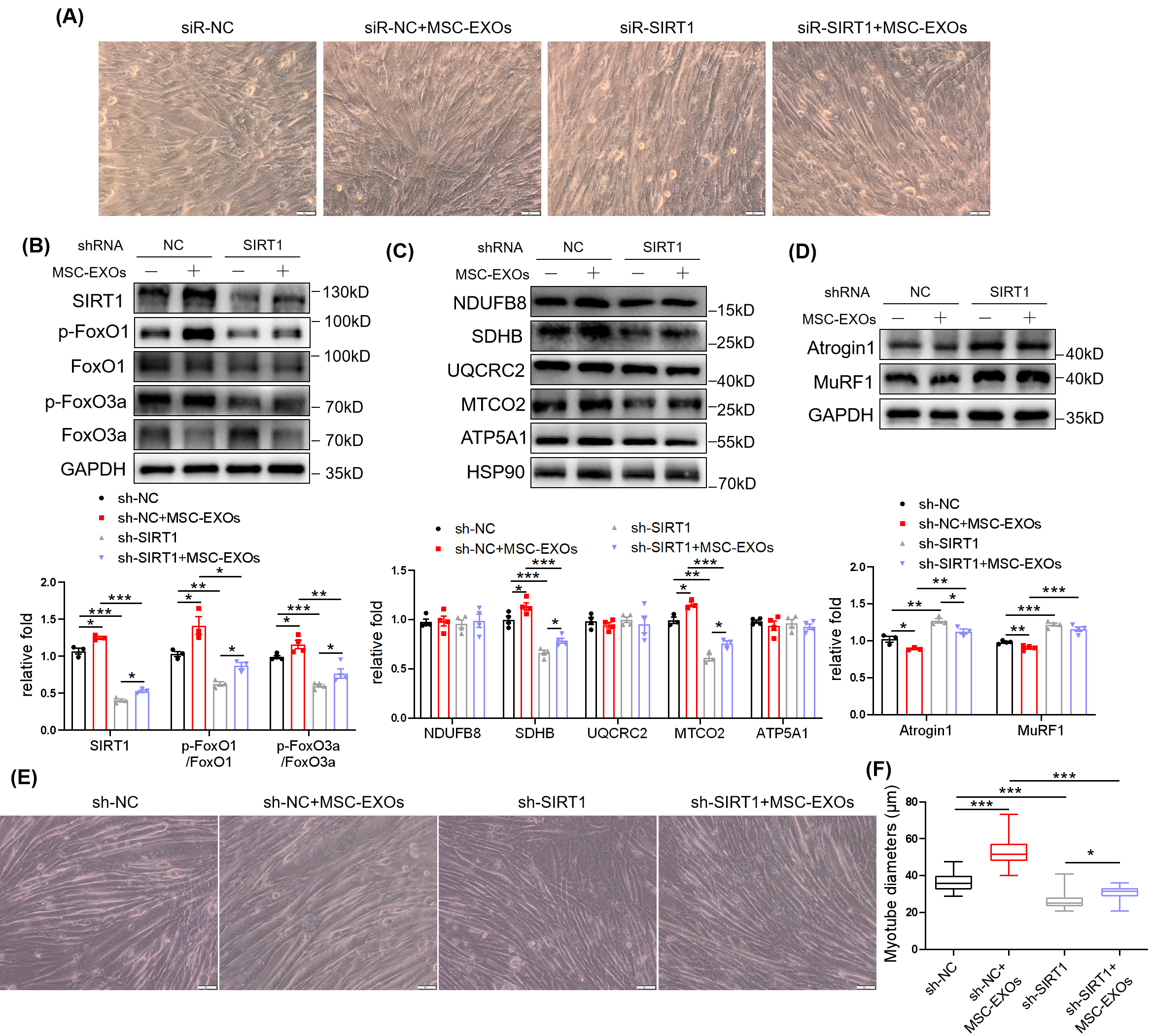
**

**Supplementary Fig. 5** MSC-EXOs counteracts myotube atrophy via enhancing SIRT1-mediated mitochondrial function. *(A)* Imaging of C2C12 myotubes transfected with SIRT1 siRNA and treated with MSC-EXOs (Scale bar, 50 μm). *(B)* Western blot analysis of SIRT1, p-FoxO1 (S319), FoxO1, p-FoxO3a (S253), and FoxO3a in C2C12 myotubes transfected with SIRT1 shRNA and treated with MSC-EXOs (n=3-4). *(C)* Western blot analysis of mitochondrial complex NDUFB8, SDHB, UQCRC2, MTCO2, and ATP5A1 (n=3–4). *(D)* Western blot analysis of Atrogin 1 and MuRF1 (n=3-4). *(E)* Imaging of C2C12 myotubes transfected with SIRT1 shRNA and treated with MSC-EXOs (Scale bar, 50 μm). *(F)* Diameters of C2C12 myotubes. Quantification of bands was performed using ImageJ software. Data are mean ± SEM. (**P* < 0.05, ***P* < 0.01, ****P* < 0.001)

.


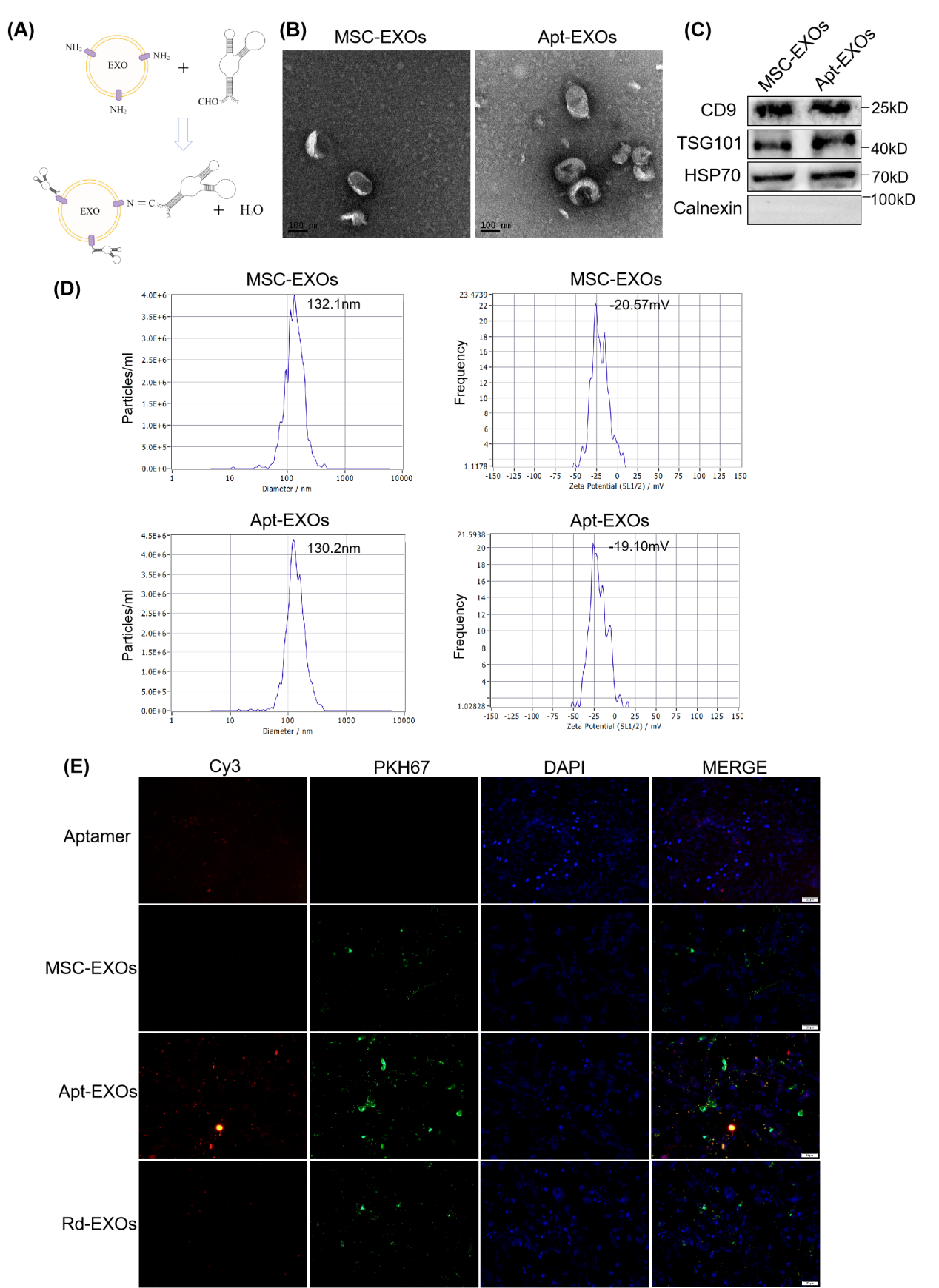


**Supplementary Fig. 6** The effects of aptamer conjugation on MSC-EXOs. *(A) C*onjugation of aptamer to MSC-EXOs via the Schiff base reaction. *(B)* TEM images of aptamer-functionalized exosomes (Apt-EXOs). *(C)* Western blot analysis of the exosomal markers CD9, TSG101, HSP70, and endoplasmic reticulum marker Calnexin of Apt-EXOs. *(D)* Nanoparticle tracking analysis of exosomal sizes. *(E)* Fluorescence-tracing of PKH67-labeled MSC-EXO/Apt-EXO uptake by C2C12 myoblasts (Scale bar, 20 μm).


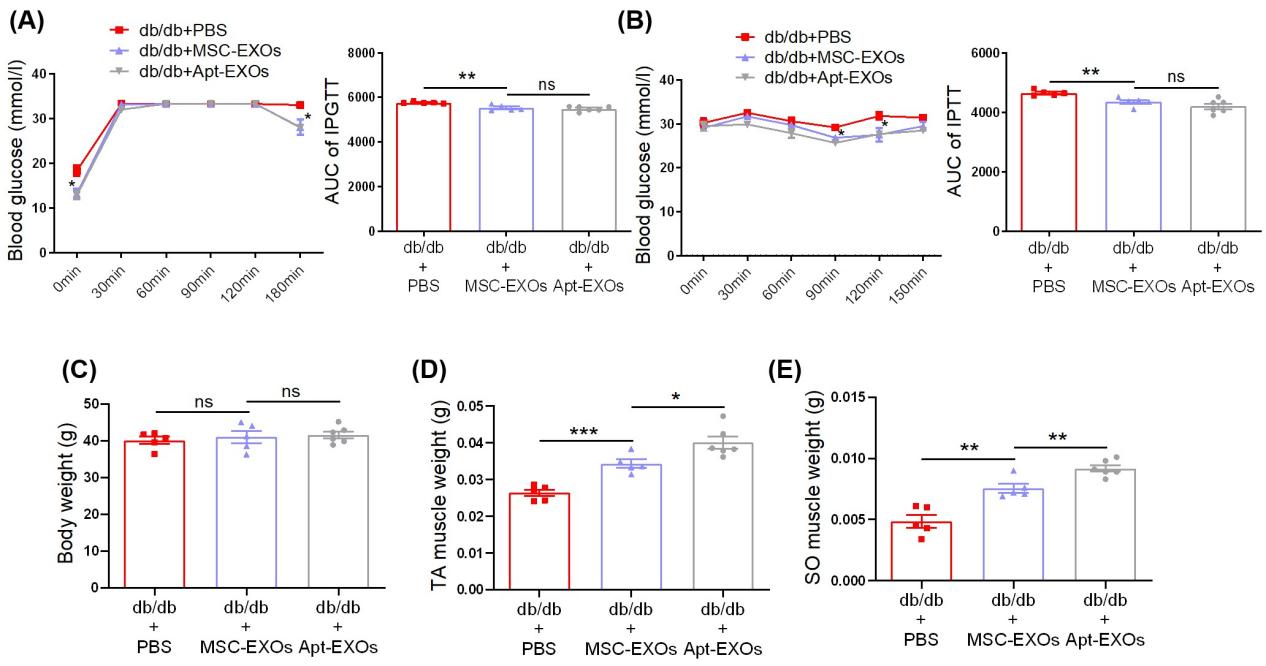


**Supplementary Fig. 7** Aptamer conjugation strengthens the effects of MSC-EXOs on muscle atrophy. *(A)* IPGTT and AUC of db/db mice after Apt-EXO injection (n = 5–6 mice). *(B)* IPITT and AUC of db/db mice after Apt-EXO injection (n = 5–6 mice). *(C)* Body weight (n = 5–6 mice). *(D)* TA muscle weight (n = 5-6 mice). *(E)* SO muscle weight (n = 5–6 mice). Data are mean ± SEM. (**P* < 0.05, ***P* < 0.01, ****P* < 0.001)
