# Supplementary material for: Aptamer‐Conjugated Exosomes Ameliorate Diabetes‐Induced Muscle Atrophy by Enhancing SIRT1/FoxO1/3a‐Mediated Mitochondrial Function
Source: J Cachexia Sarcopenia Muscle. 2025 Jan 28;16(1):e13717. doi: 10.1002/jcsm.13717 (PMC11773161; doi:10.1002/jcsm.13717)
Supplement: Supplementary file 2 — Data S1 Supplementary Information. [file JCSM-16-e13717-s002.docx]

**Supplementary References**

[S1]Bonnard C, Durand A, Peyrol S, [Chanseaume](https://pubmed.ncbi.nlm.nih.gov/?term=Chanseaume+E&cauthor_id=18188455) E, [Chauvin](https://pubmed.ncbi.nlm.nih.gov/?term=Chauvin+MA&cauthor_id=18188455) MA, [Morio](https://pubmed.ncbi.nlm.nih.gov/?term=Morio+B&cauthor_id=18188455) B, et al. Mitochondrial dysfunction results from oxidative stress in the skeletal muscle of diet induced insulin-resistant mice. The Journal of clinical investigation. 2008;118:789-800.
[S2]Cho JE, Fournier M, Da X, [Lewis](https://pubmed.ncbi.nlm.nih.gov/?term=Lewis+MI&cauthor_id=19850732) MI. Time course expression of Foxo transcription factors in skeletal muscle following corticosteroid administration. J Appl Physiol. 2010;108:137–145.
[S3]Sun Y, Shi H, Yin S, [Ji](https://pubmed.ncbi.nlm.nih.gov/?term=Ji+C&cauthor_id=30052036) C, [Zhang](https://pubmed.ncbi.nlm.nih.gov/?term=Zhang+X&cauthor_id=30052036) X, [Zhang](https://pubmed.ncbi.nlm.nih.gov/?term=Zhang+B&cauthor_id=30052036) B, et al. Human Mesenchymal Stem Cell Derived Exosomes Alleviate Type 2 Diabetes Mellitus by Reversing Peripheral Insulin Resistance and Relieving beta-Cell Destruction. ACS nano. 2018,12: 7613-7628.

[S4]Tenchov R, Sasso JM, Wang X, Liaw WS, Chen CA, Zhou QA. [Exosomes─Nature's Lipid Nanoparticles, a Rising Star in Drug Delivery and Diagnostics.](https://pubmed.ncbi.nlm.nih.gov/36354238/) ACS Nano. 2022,16(11):17802-17846.

[S5]Sadeghi S, Tehrani FR, Tahmasebi S, Shafiee A, Hashemi SM. Exosome engineering in cell therapy and drug delivery. Inflammopharmacology. 2023;31(1):145-169.

[S6]Ueki R, Atsuta S, Ueki A and Sando S. Nongenetic Reprogramming of the Ligand Specificity of Growth Factor Receptors by Bispecific DNA Aptamers. J. Am. Chem. Soc. 2017, 139, 6554–6557.

[S7]Yang Y, Zhang J, Wu S, Deng Y, Wang S, Xie L, et al. [Exosome/antimicrobial peptide laden hydrogel wound dressings promote scarless wound healing through miR-21-5p-mediated multiple functions.](https://pubmed.ncbi.nlm.nih.gov/38581764/) Biomaterials. 2024,308:122558.

[S8]Sengupta V, Sengupta S, Lazo A, Woods P, Nolan A, Bremer N. [Exosomes Derived from Bone Marrow Mesenchymal Stem Cells as Treatment for Severe COVID-19.](https://pubmed.ncbi.nlm.nih.gov/32380908/) Stem Cells Dev. 2020;29(12):747-754.

[S9]Shen S, Liao Q, Liu J, Pan R, Lee SM, Lin L. Myricanol rescues dexamethasone-induced muscle dysfunction via a sirtuin 1-dependent mechanism. J Cachexia Sarcopenia Muscle. 2019;10(2):429-444.
